# CDK4/6 Inhibitors in Breast Cancer Treatment: Potential Interactions with Drug, Gene, and Pathophysiological Conditions

**DOI:** 10.3390/ijms21176350

**Published:** 2020-09-01

**Authors:** Rossana Roncato, Jacopo Angelini, Arianna Pani, Erika Cecchin, Andrea Sartore-Bianchi, Salvatore Siena, Elena De Mattia, Francesco Scaglione, Giuseppe Toffoli

**Affiliations:** 1 Experimental and Clinical Pharmacology Unit, Centro di Riferimento Oncologico (CRO), IRCCS, 33081 Aviano, Italy; ececchin@cro.it (E.C.); edemattia@cro.it (E.D.M.); gtoffoli@cro.it (G.T.); 2 Department of Oncology and Hemato-Oncology, Università degli Studi di Milano, 20122 Milan, Italy; jacopoangelini1@gmail.com (J.A.); arianna.pani@unimi.it (A.P.); andrea.sartorebianchi@ospedaleniguarda.it (A.S.-B.); salvatore.siena@ospedaleniguarda.it (S.S.); francesco.scaglione@unimi.it (F.S.); 3 Clinical Pharmacology Unit, ASST Grande Ospedale Metropolitano Niguarda, Piazza dell’Ospedale Maggiore 3, 20162 Milan, Italy; 4 Department of Hematology and Oncology, Niguarda Cancer Center, Grande Ospedale Metropolitano Niguarda, 20162 Milan, Italy

**Keywords:** cyclin-dependent kinases inhibitors, breast cancer, personalized medicine

## Abstract

Palbociclib, ribociclib, and abemaciclib belong to the third generation of cyclin-dependent kinases inhibitors (CDKis), an established therapeutic class for advanced and metastatic breast cancer. Interindividual variability in the therapeutic response of CDKis has been reported and some individuals may experience increased and unexpected toxicity. This narrative review aims at identifying the factors potentially concurring at this variability for driving the most appropriate and tailored use of CDKis in the clinic. Specifically, concomitant medications, pharmacogenetic profile, and pathophysiological conditions could influence absorption, distribution, metabolism, and elimination pharmacokinetics. A personalized therapeutic approach taking into consideration all factors potentially contributing to an altered pharmacokinetic/pharmacodynamic profile could better drive safe and effective clinical use.

## 1. Introduction

**Figure 1:**
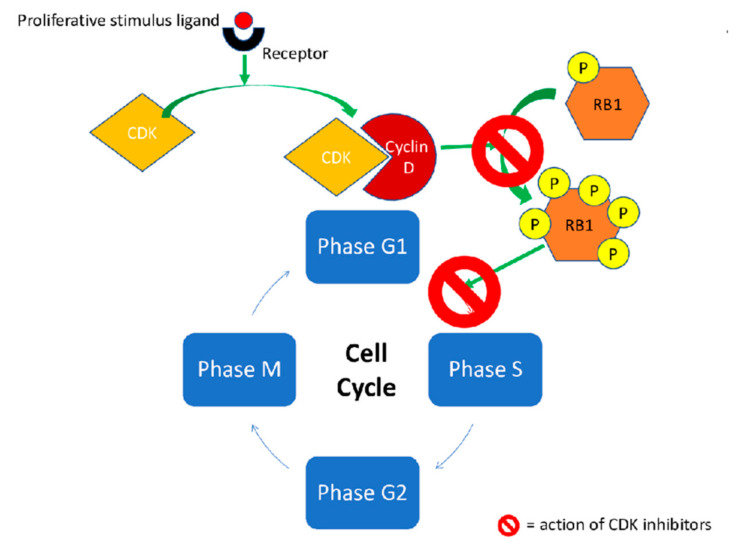
In red the enzymatic reactions prevented by CDK inhibitors are shown. Physiologically, growth signals induce the expression of Cyclin D which binds CDK4 or CDK6; this CDK-Cyclin complex promotes the hyper-phosphorylation of the tumor suppressor protein RB1 which is bound to the E2F transcription factor, determining its release and the consequent expression of genes implicated in cellular entry in S phase and proliferation, as topoisomerase-IIα. The hyper-expression of Cyclin D is shown in hyper-activated cellular signaling, involved in the oncogenesis of several tumors, as RAS/RAF/MEK/ERK and PI3K/AKT/mTOR pathways. When CDK inhibitors are used, the hyper-phosphorylation of the RB1 by complex CDK-Cyclin D and the consequent transcription of important enzymes allowing the transition from G1 to S phase of cell-cycle, such as topoisomerase-Iiα, are prevented. CDK—Cyclin Dependent Kinase; RB1—RB transcriptional corepressor 1; P—Phosphate group.

The complexes composed by cyclins and cyclin-dependent kinases (CDKs) are critical checkpoints in the progression of the cell cycle and some mutations on this pathway have been demonstrated to be involved in cancer [1]. In breast cancer, CDK4 and CDK6 inhibitors (CDKis) have emerged as key players in the cellular proliferation through different pathways [2,3]. The mechanism of action of CDKis is shown in Figure 1.

Breast cancer represents an interesting pathological setting where CDKis constitute a current therapeutic tool and also provide the most advanced perspectives, especially in advanced hormone receptor (HR)-positive (estrogen receptor-positive and/or progesterone receptor-positive) and human epidermal growth factor receptor 2 (HER2)-negative metastatic breast cancer [2,4,5].

The EMA and FDA have approved palbociclib, ribociclib, and abemaciclib in combination therapy with aromatase inhibitors (anastrozole/letrozole) or fulvestrant. In this clinical setting, CDKis used as first-line therapy showed an improvement of progression-free survival (PFS) from 14–16 months in the placebo arms to ≥25 months in the experimental arm with palbociclib, ribociclib or abemaciclib as demonstrated in their registration trials: PALOMA-2, MONALEESA-2, and MONARCH-3 respectively [6,7,8]. In the MONALEESA-7 trial ribociclib showed similar efficacy in combination with ovarian suppressor and tamoxifen or aromatase inhibitor at the same setting in premenopausal women [9]. Nevertheless, regulatory agencies do not approve the combination ribociclib and tamoxifen, due to safety reasons related to increased QT prolongation [10,11]. When CDKis are used in second-line therapy, due to relapse on aromatase inhibitors, they doubled PFS in combination with fulvestrant compared to fulvestrant alone, as demonstrated in PALOMA-3, MONALEESA-3, and MONARCH-2 [12,13,14].

FDA approved abemaciclib also as monotherapy for the treatment of adult patients with HR-positive, HER2-negative advanced or metastatic breast cancer with disease progression following endocrine therapy and prior chemotherapy in the metastatic setting. On the contrary, EMA did not approve abemaciclib in monotherapy considering that MONARCH-1 is a phase II single-arm open-label study, characterized by heterogeneous study population, due to wide inclusion criteria (women who had progressed on or after prior endocrine therapy and had one or two chemotherapy regimens) [15,16]. Furthermore, the post-marketing observational data from the Flatiron Health database were used as historic cohort to compare MONARCH-1 data, but different endpoints between the two studies make them not directly comparable and no robust clinical evidence was assessed by EMA to support the approval of abemaciclib as monotherapy [16].

Palbociclib, ribociclib and abemaciclib share the same mechanism of action and show similar pharmacokinetic (PK) and pharmacodynamic (PD) profiles. Nonetheless, some discrepancies in their PK and CDKs isoforms selectivity need to be outlined. It can account for different response to treatment highlighted in some studies [17]. Differently from registration trials, in clinical practice all factors that may interfere with PK/PD and toxicological properties of investigated drugs and related to concomitant epidemiological, genetic and clinical conditions should be taken in consideration by clinicians. In this regard, a meta-analysis of clinical trials involving CDKis in combination with aromatase inhibitors or tamoxifen suggests a higher absolute prevalence of some adverse events in a subgroup composed by asiatic patients compared with the non-Asian group. A significantly different efficacy profile was also found in the same patients’ subgroup [18,19]. This phenomenon has been proposed to be ascribable not only to the different epidemiology features of breast cancer in Asiatic patients, but also to specific multifactorial causes. In particular, they concern: a) differences in body size, which can result a critical issue due to the fixed-dose schedule of these drugs [20,21]; b) diet, where relevant intake of soy influences drugs metabolism [22]; c) a different expression of proinflammatory genes involving the oncogenesis; d) genetic background of enzymes involved in CDKis absorption, distribution, metabolism, and elimination (ADME), as suggested by higher average C_max_ and AUC values of ribociclib in Japanese patients [19,23,24]. In our opinion, this latter point suggests a key-role of both cytochromes and transporters in influencing the ADME of CDKis PK and consequently their own tolerability profiles.

The aim of this review is to discuss possible meaningful clinical interactions between CDKis and co-administered drugs (drug-drug interactions, DDIs), pharmacogenetics (drug-gene interactions, DGIs) and pathophysiological conditions that may occur at all levels of ADME. The differences in CDKis pharmacological features could be further exacerbated by decreasing or increasing their oral bioavailability, distribution, and/or toxicity. With regard to all these aspects, not considered in CDKis phase III-IV trials, we aim to focus on patient’s features (DDIs, DGIs, and pathophysiological conditions) to foster a more personalized use of these agents [25].

### 1.1. Pharmacodynamics

Palbociclib, ribociclib and abemaciclib selectively and reversibly inhibit CDK4/6 binding to ATP pocket of the inactive complex by two hydrogen bonds. In enzymatic assays, each compound shows a different potency in the activity against CDK4 or CDK6. Palbociclib shows a similar activity for CDK4 and CDK6 (CDK4 IC_50_ = 11 nmol/L vs. CDK6 IC_50_ = 16 nmol/L), while ribociclib potency is higher for CDK4 than for CDK6 (CDK4 IC_50_ = 10 nmol/L vs. CDK6 IC_50_ = 39 nmol/L) [26]. Abemaciclib is the most potent inhibitor, especially toward CDK4 (CDK4 IC_50_ = 2 nmol/L vs. CDK6 IC_50_ = 10 nmol/L) [26,27,28,29]. Abemaciclib, by virtue of its functional group, achieves a better steric complementarity in the CDK4/6 ATP cleft compared to palbociclib and ribociclib [30]. Besides presenting a higher potency toward CDK4 inhibition, abemaciclib, when higher concentration is achieved, shows the least selectivity against CKDs-cyclins complex compared to palbociclib and ribociclib, as additional interactions occur with other redundant CDK isoforms (Table 1) [31,32,33]. Hence, abemaciclib conducts its main efficacy effect targeting CDK4, and secondly, with a lower potency, CDK9 and to an even lesser extent CDK6, responsible for suppressing RB1 phosphorylation and the cell cycle arrest in the G1 phase overall [2,16].

Interestingly, abemaciclib, independently from CDKs pathways, presents multiple mechanisms of action compared to palbociclib and ribociclib, inhibiting other kinases that are involved in many processes influencing cellular proliferation, inflammation, and oncogenesis (Table 1).

### 1.2. Safety

By virtue of CDKis PD properties and CDK activity in cell-cycle, palbociclib, ribociclib and abemaciclib share a common toxicity profile against high proliferative tissue (e.g., bone marrow and gastrointestinal mucosa), consisting in hematological and gastrointestinal disorders such as anemia, neutropenia, white blood cells decrease, nausea, vomiting and diarrhea [11,16,35]. Nevertheless, palbociclib, ribociclib and abemaciclib show a better efficacy and safety profile compared with the first and the second generations of CDK inhibitors, which instead target all or most of CDK isoforms [36]. This promiscuity toward CDK isoforms often resulted in a jeopardizing inability to discriminate physiological and malignant proliferative processes, inducing severe cytotoxicity (myelosuppression and anemia) [36]. Through an increased selectivity, new CDKis show less common and severe hematological adverse events [31]. Gastrointestinal disorders are instead the most frequent adverse events recorded for abemaciclib with higher incidence of nausea and severe diarrhea compared with palbociclib and ribociclib. For these reasons abemaciclib should not be recommended in patients with gastrointestinal comorbidities [16,37,38]. The different abemaciclib tolerability profile could be attributed to the main involvement of CDK9, compared to CDK6 which is remarkably implicated in hematopoiesis [37]. Consequently, myelosuppression and anemia are less frequent, and neutropenia is quite easily manageable, compared to palbociclib and ribociclib. Furthermore, the safer abemaciclib profile compared with first and second generation CDK inhibitors could be ascribed to its lower potency against CDK1, CDK7, and CDK9, overall [31]. Interestingly, abemaciclib-induced diarrhea can significantly reduce its absorption, so that a prophylactic dose of loperamide 8 mg could be usually recommended [3].

A very relevant safety issue concerns ribociclib and arrhythmias. In fact, ribociclib in phase III trials has been associated to QT interval prolongation, distinguishing from palbociclib and abemaciclib [11]. A recent pooled safety analysis (1065 patients treated with ribociclib vs. 818 patients with placebo) shows that the QT interval corrected for heart rate (QT-c) and calculated by the Fridericia formula (QTcF) >480 ms occurred in 5% vs. 1% of patients in the ribociclib vs. placebo arms, whereas a QTcF >500 ms occurred in 1% vs. <1% of patients respectively [39]. On the contrary, no relevant adverse event related to prolonged QT is reported for palbociclib and abemaciclib [40,41]. For this reason, ribociclib should be avoided in case of patients suffering from relevant cardiological disease (bradyarrhythmia, long QT syndrome, recent ischemic myocardial syndrome, heart failure and electrolyte abnormalities) or assuming concomitant medications directly or indirectly inducing QT prolongation. For patients starting with ribociclib, an electrocardiography (ECG) monitoring is required in order to observe electrophysiological consequences [11]. Palbociclib and abemaciclib, differently from ribociclib, have no direct QT interval influence, nevertheless the co-administration of drugs that prolong QT is discouraged [16,35].

A summary of main safety issues recorded in registration trials of each compound is reported in Table 2. Post-registration safety data warn against the increased risk of venous thromboembolism (VTE) and pulmonary embolism in patients treated with abemaciclib, as reported in abemaciclib leaflet [16]. Nevertheless, increasing evidences stress on the higher risk of VTE for all the aforementioned CDKis than the placebo group, suggesting caution and additional monitoring of patients on treatment with CDKis [42]. For a possible rationale for CDKis selection according to pathophysiological conditions, see Section 7.

### 1.3. Pharmacokinetics

Oral agents are usually characterized by a far greater PK variability than intravenous agents [43]. Regardless CDKis are oral drugs characterized by a similar PK profile, palbociclib and ribociclib show different features from abemaciclib. These peculiarities influence their therapeutic use in terms of schedule of administration and dose adjustments, according to PK details reported in Table 3. Palbociclib and abemaciclib are slowly absorbed [44], whereas ribociclib is more rapidly absorbed compared with palbociclib and abemaciclib [39].

Palbociclib, ribociclib, and abemaciclib are substrates of P-glycoprotein (P-gp; ABCB1) and breast cancer resistance protein (BCRP; ABCG2). They are both efflux transporter proteins which are localized in important anatomical structures such as blood brain barrier (BBB), proximal tubule cells, enterocytes, and hepatocytes [50]. These transporters play an important role in the first-pass elimination of orally administered drug influencing their bioavailability in the intestine by (a) effluxing them at the lumen-facing epithelia of the small intestine and colon, and the bile-facing canaliculi of the liver, (b) excreting from the systemic circulation at the urine-facing side of the brush border membrane of proximal tubules in the kidney and by (c) modifying their therapeutic concentration in different target tissues as in BBB. In particular, in vitro studies suggest that palbociclib and abemaciclib are substrates of P-gp and BCRP at the BBB, affecting their passage through, while ribociclib is mainly substrate for intestinal P-gp, which may possibly affect its oral absorption rate rather than its brain concentration [50,51,52]. Regardless, recent data from in vivo experimentation suggest for ribociclib a more similar profile to other CDKis as substrate of P-gp at the BBB [53]. In particular, due to the peculiar lipophilicity properties, palbociclib and abemaciclib easily penetrate the BBB, but compared to palbociclib and ribociclib only abemaciclib achieves and maintains the therapeutic concentration at lower doses [51,54]. This favorable profile in central nervous system (CNS) can be also explained by the dose-related inhibiting effect of abemaciclib on P-gp and BCRP. On the contrary, palbociclib [51] and, from recent in vivo evidences, ribociclib [53] appears to be abundantly removed from cerebrospinal fluid. In vitro data suggest also that palbociclib and ribociclib may inhibit intestinal P-gp and BCRP with a possible increase in the drug absorption into the bloodstream. No evidence of such an interaction is available in vivo [50].

Inter- and intra-patient variability after administration of CDKis represents a relevant issue for these compounds, especially for ribociclib [11,55]. Ribociclib is not influenced by food intake nor gastric pH changes, differently from palbociclib and abemaciclib [55]. Palbociclib variability has been reported to be reduced on fed state [11,56]. In the registration clinical study [45], 13% of all patients with palbociclib administration under fasting conditions revealed a lower palbociclib exposure. A study conducted on healthy volunteers showed a higher C_max_ in subjects who assumed palbociclib with a high fat meal. This is probably due to the dissolution of palbociclib capsule that is highly dependent on pH with an inverse relationship. Abemaciclib is characterized by an extensive variability in cancer patients, independently from demographics (sex, age, body weight). Data from healthy subjects show that increasing the dose and/or duration of abemaciclib treatment results in a reduction in the fraction of dose absorbed [57]. Food intake slightly modifies abemaciclib PK profile without relevant clinical consequences; although high fat and high caloric meal increases AUC by 9% and C_max_ by 26%. Overall PK variability of abemaciclib is not meaningfully influenced by food-effect [16,57].

Palbociclib and ribociclib have a moderate binding to human plasma proteins and a high volume of distribution (Vd) [49], on the contrary abemaciclib is highly bound to plasma proteins and thus it is characterized by a lower Vd.

All three drugs undergo hepatic metabolism, being metabolized by the CYP3A4 [50]. Ribociclib is characterized by the longest half-life among CDKis and its toxicity profile prevents a continuous schedule [29,58] as also for the case of palbociclib [45]. Abemaciclib can be instead administered continuously [2,15,57,59].

Palbociclib has shown a proportional dose accumulation, influencing C_max_ and AUC values and has a dose-independent PK when administered in a single dose. Since it is a weak time-dependent inhibitor of a CYP3A4, palbociclib PK is expected to be dose dependent in multiple dose conditions, due to the inhibition of its own metabolism. On the contrary, ribociclib has a non-linear PK and is a strong CYP3A4/5 time-dependent inhibitor when administered at a 600 mg dose, and a moderate CYP3A4 inhibitor at a 400 mg dose. Ribociclib reversibly inhibits also CYP1A2 and CYP2E1. Abemaciclib and its major active metabolites have been proven to downregulate mRNA of CYPs, including CYP1A2, CYP2B6, CYP2C8, CYP2C9, CYP2D6, and CYP3A4.

Palbociclib, ribociclib, and abemaciclib have not been studied in pregnancy, but several in vivo studies show teratogenic issues and their use is not recommended in this clinical setting and during lactation [11,16,46].

## 2. Potential Drug-Drug Interactions

### 2.1. Potential Drug-Drug Interactions with ADME

Hereinafter the potential DDIs based on the most common comorbid conditions will be summarized. Meaningful clinical drug interactions have been extensively described, according both to the results from the consensus workshop [50] on medication that can be safely administered concomitantly with palbociclib, ribociclib and abemaciclib and to each FDA leaflet [10,45]. DDIs with abemaciclib have been described using the information contained in the leaflet [46] and by analogy with the other CDKis. The list of all drugs major substrate for CYP3A4, sensitive or with narrow therapeutic index (NTI), is reported in Table S1.

#### 2.1.1. Agents That May Alter CDK4/6 Inhibitors Absorption

Drug absorption from the gut could be source of intra- and inter-patient variability as well as PK variability. Absorptive surface area, transit time through the gut, blood flow to the site of absorption, and gastric and intestinal pH are among the factors influencing the absorption [43].

(1) Gastric pH Elevating Medications

The most frequent interactions affecting absorption, and thus drug bioavailability, are due to gastric pH modification perpetrated by proton pump inhibitor (PPIs), H2-receptor antagonists or local antacids [50]. The effect of acid-reducing agents on CDKis was only studied in palbociclib. Two clinical trials performed in healthy subjects found that co-administration of a single dose of palbociclib with multiple doses of the PPI rabeprazole decreased palbociclib C_max_ by 41% and 62% under fed and fasted conditions, respectively. The AUC decrease was instead relevant only in the fasted state (80% vs. 13% in the fed state). Thus, considering that local antacids and H2-receptor antagonists are less efficacious than PPIs in reducing gastric acidity, the effect of drug elevating gastric pH is expected to be minimal on fed state. Nonetheless, concomitant use PPIs should be avoided with palbociclib. No DDIs are expected with PPIs, H2-receptor antagonists, or antacids with ribociclib and abemaciclib that can be taken with or without food.

(2) Membrane Transporters

Besides being inhibitors, ribociclib and abemaciclib are also substrate of P-gp and BCRP while palbociclib it is not in most tissue (except for BBB). DDIs are expected from competition with other substrates for these membrane transporters. However, as reported in Section 2.1.4, guidelines on management of such interactions are still lacking.

#### 2.1.2. Agents That May Alter CDK4/6 Inhibitors Distribution

Plasma protein binding can have multiple effects on both PK and PD of a drug. Only free drug can produce the therapeutic effect because it can be distributed to the site of action and interact with receptors. Competitive displacements are the predominant interactions among protein binding reactions that results in increased free plasma concentration of the displaced drug. Because palbociclib and ribociclib are not highly protein bound (palbociclib 85% and ribociclib 70%) distribution related DDIs are not expected. Regardless, the mean bound fraction of abemaciclib was found to be 96.3%, 93.4% for M2, 96.8% for M18, and 97.8% for M20, its active metabolites. For drugs highly bound to plasmatic proteins, displacement reactions may acquire clinical importance. Future studies are warranted to evaluate and monitor the effects of these potential DDIs.

#### 2.1.3. Agents That May Alter CDK4/6 Inhibitors Metabolism

Metabolism of all CDKis could be impacted to a various extent by strong to moderate inhibitors and inducers of CYPs of which they are substrate. Palbociclib, ribociclib, and abemaciclib undergo extensive CYP3A-mediated hepatic metabolism.

(1) CYP3A Inhibitors May Increase CDK4/6 Inhibitors Plasma Concentrations

In the FDA leaflet it is recommended to avoid the concomitant use of strong CYP3A inhibitors (e.g., clarithromycin, protease inhibitor for HIV and HCV, itraconazole, ketoconazole, posaconazole, voriconazole, ritonavir, saquinavir, grapefruit) because of the increase in the recorded CDKis plasma exposure that may lead to increased toxicity [10,45,46]. Particularly, plasma exposure of abemaciclib and its active metabolites is increased to a clinically meaningful extent when administered with strong CYP3A inhibitors. Strong CYP3A4 inhibitors when co-administered with CDKis may also lead to an enhanced risk for prolonged QT interval as well (see Section 2.2.). If an alternative with less potential for CYP3A inhibition could not be considered, the dosage of the CDKis needs to be reduced to 75 mg/day (↓40%) for palbociclib, to 400 mg/day (↓33.33%) for ribociclib and to 100 mg twice a day (↓50/33.33%) for abemaciclib. In patients who have had a dose reduction to 100 mg twice daily due to adverse reactions, the abemaciclib dose should be further reduced to 50 mg twice daily when concomitantly used with other strong CYP3A inhibitors. If the strong inhibitor is discontinued, after five half-lives of the inhibitor, the dose of CDKis could be increased to the dosage used prior the introduction of the inhibitor. With regard to moderate inhibitors (e.g., erythromycin, ciprofloxacin, fluconazole, isavuconazole, aprepitant, netupitant, nifedipine, nicardipina, verapamil, ziprasidone) a risk of increased CDKis exposure is possible, although, at present only data regarding abemaciclib and ribociclib are available (see Table 3). In the FDA leaflet, it is recommended to monitor a possible increased toxicity, while for weak inhibitors (e.g., ticagrelor, cilostazol, fosaprepitant, alprazolam), only a low risk of increased exposure is reported [10,45,46]. Dose adjustment suggestions are summarized in Table 4.

Available data on changes in PK parameters in healthy subjects for each CDKi following co-administration with strong and moderate inhibitors are reported in Table 3, while the most used CYP3A inhibitors were reviewed and classified by drug interaction risk potential in Table S1.

(2) CYP3A Inducers May Decrease CDK4/6 Inhibitors Plasma Concentrations

Co-administration of strong CYP3A4 inducers decreases CDKis plasma exposure and may lead to reduced activity and drug failure. As CYP3A strong inhibitors, strong inducers (e.g., phenytoin, rifampin, carbamazepine, phenobarbital, and St John’s Wort) should be avoided and an alternative with less potential to induce CYP3A4 is recommended. The effect of moderate CYP3A inducers (e.g., efavirenz) on the PK of CDKis has been studied only on palbociclib and ribociclib. It is recommended in the FDA leaflet to monitor a possible increased risk of decreased exposure and a lack of efficacy [10,45,46]. There is a low risk of decreased exposure of CDKis for co-administration with weak inducers (e.g., nevirapine, dexamethasone, oxcarbazepine, clobazam).

Available data on changes in PK parameters recorded in healthy subjects for each CDKi following co-administration with strong and moderate inducers are reported in Table 3, while the most commonly used CYP3A inducers were reviewed and classified by potential drug interaction risk in Table S1.

#### 2.1.4. Agents That May Be Altered by Co-Administration with CDK4/6 Inhibitors

When CYP3A4 major substrates are concomitantly administered with CDKis, their serum concentration could be increased by virtue of CDKis inhibitory potential on CYP3A4, and a dose reduction may be warranted especially with ribociclib (see Section 1.3).

Caution is recommended especially when the co-administered drugs besides being CYP3A4 major substrate are also characterized by a NTI and/or are sensitive substrate drugs where small variations of drugs can cause toxicity (e.g., alfentanil, cyclosporine, dihydroergotamine, ergotamine, everolimus, fentanyl, pimozide, quinidine, sirolimus, tacrolimus, simvastatin, and atorvastatin; see Table S1). Sensitive substrates are defined by FDA as substrates that can induce a five-fold or greater increase in AUC with strong index inhibitors while moderately sensitive substrates, when the increase in AUC is two-fold or greater to less than five-fold. Co-administration of midazolam in healthy subjects with multiple dose of either palbociclib or ribociclib 400 mg and 600 mg increased midazolam plasma exposure of 61%, 3.8-fold and 5.2-fold, respectively, compared to the administration of midazolam alone [10,45]. Caution may be necessary also when abemaciclib, highly protein bound, is co-administered with drug with NTI. The displacer drug may produce a rapid increase in plasma concentration of the displaced medication possibly leading to enhanced pharmacological effect and/or toxicity. Nevertheless, there are no guidelines on how to tackle these types of interactions.

The list of all drugs major substrate for CYP3A4, sensitive or NTI for which a dose adjustment could be considered according to Bellet et al. [50], is shown in Table S1.

Other DDIs described with CDKis results from competition for the membrane transporters. Caution should be exercised for CDKis co-administration with metformin, which is a relevant substrate of renal OCT2, MATE1, and MATE2-K transporters because of the risk of reduced renal clearance and secretion of metformin. Furthermore, at clinically relevant concentrations, it has been shown that palbociclib in vitro potentially inhibits intestinal OCT1 along with intestinal P-gp and BCRP. Moreover, ribociclib potentially inhibits OATP1B1/B3, OCT1, OCT2, BSEP, and MATE1 along with P-gp and BCRP, while abemaciclib and its major active metabolites inhibit OCT2, MATE1, and MATE2-K, along with P-gp and BCRP. Although no data are available yet, it may be hypothesized that, at therapeutic doses, CDKis enhance toxicity risk of drugs that are substrates for P-gp due to the inhibition of the efflux pump and with a consequent increase in their plasmatic concentration.

#### 2.1.5. Pain Killer (Opioids and NSAIDs)

According to the International Association for the Study of Pain, pain prevalence in breast cancer varies from 40% to 89%. Pain control represents indeed one of the greatest challenges in patients with cancer. The most commonly used drugs for pain control were reviewed and classified by drug interaction risk potential in Table S1. Reported high risk DDIs involve major CYP3A4 substrate/NTI drugs.

### 2.2. Potential Drug-Drug Interactions with Non ADME Agents That May Potentiate CDK4/6 Inhibitors Toxicity

The meaningful DDIs that will be hereinafter reviewed concern CDKis mechanism of action rather than their ADME profile. As mentioned in our Safety Section 1.2., ribociclib has a special warning for electrophysiological toxicity, suggesting a careful monitoring and a dose interruption, discontinuation, or a dose modification [11]. Drugs with a known potential to prolong QT such as antiarrhythmic medicines (including, but not limited to amiodarone, disopyramide, procainamide, quinidine and sotalol) and other drugs that are known to prolong the QT interval (including, but not limited to, chloroquine, halofantrine, clarithromycin, haloperidol, methadone, moxifloxacin, bepridil, pimozide and ondansetron) could also increase the risk of palbociclib and abemaciclib for QT prolongation interval.

Differently from palbociclib and abemaciclib, MONALEESA-2 trial showed that patients assuming ribociclib reported more events of QT prolongation, independently from concomitant therapies [60]. In this regard, Table S1 reports a differential risk for prolonged QT interval deriving from DDIs between palbociclib, abemaciclib and ribociclib.

#### Antidepressant

Antidepressant treatment is often useful in patients with breast cancer to treat anxiety and stress-related disorders and is also indicated for specific symptoms such as insomnia (mirtazapine and trazodone), neuropathic pain (duloxetine and amitriptyline), hot flashes (venlafaxine and SSRIs), lack of appetite, nausea and/or vomiting (mirtazapine) and fatigue (bupropion and methylphenidate). A major concern is the effect of antidepressants on QTc interval prolongation and the risk of Torsade de Pointes (TdP). Citalopram and escitalopram prolong QTc and present known risk of TdP, while mirtazapine and venlafaxine with possible risk for TdP. DDIs with CDKis exist for most antidepressants, either via CYP3A4 or mostly via QTc prolongation.

The most commonly used antidepressants were reviewed and classified by drug interaction risk potential in Table S1.

### 2.3. Other DDIs

#### 2.3.1. Osteoporosis Treatment (Denosumab, Vit D)

Denosumab is not expected to alter the PK of medicinal products metabolized by CYP3A4. Vitamin D supplementation could play a role considering that when systemic (or even local) VitD3 levels fluctuate, the disposition of the drugs that serve as CYP3A4 substrates may be changed [61].

#### 2.3.2. Potential SULT2A1-Mediated DDIs with CDK4/6 Inhibitors

Even though sulfotransferases (SULTs) enzymes are involved in the metabolism of many xenobiotics, there are few documented examples of DDIs involving these enzymes, also considering the overlapped substrate selectivity reported among different isoforms of SULTs enzymes [62].

Some drugs have demonstrated to regulate *SULT* gene expression by interacting with members of the nuclear receptor (NR) superfamily (i.e., methotrexate through interaction with constitutive androstane receptor, CAR; genistein through liver X receptor, LXR; estradiol through estrogen receptor alpha, ERα; dexamethasone by activating pregnane X receptor transcription factor (PXR) and fibrates as proliferator-activated rector alpha agonists, PPARα agonists). This could lead to an increased first-pass effect with consequent reduction in drug levels and possible loss of efficacy [62,63].

SULT2A1 is inhibited in vitro by testosterone, clomiphene, danazol, spironolactone, cyproterone and chlorpromazine. Inhibition of SULTs may reduce metabolism of drugs, leading to increased drug levels and possible toxicity [62,63].

The non-steroidal anti-inflammatory drug (NSAID) colecoxib has shown to modulate the activity of SULT2A1 by causing product switching for estradiol sulfonation and increasing overall estrogen sulfonation with a potential benefit for breast cancer patients in reducing active estrogen [62]. Potential SULT2A1-mediated DDIs with CDKis are of uncertain clinical relevance nor are supported by strong scientific evidences.

## 3. Potential Drug-Gene Interactions with ADME

All CDKis are metabolic substrates for CYP3A enzymes, CYP3A4 and CYP3A5 and consequently their pharmacogenomic profile assessment could be an important factor to be considered along with DDIs. Moreover, they are transported via ATP-Binding Cassette (ABC) transporters that were reported to affect their absorption, elimination, and distribution in the body compartments. Several single-nucleotide polymorphisms (SNPs) have been identified in the genes encoding for CYP3A4, CYP3A5 and P-gp to contribute in drug metabolism. Pharmacogenomic is the discipline studying patients’ SNPs and their relationship with the therapy outcome and is one of the approaches used to address the issue of inter-patient variability. The study of the genetic variability in ADME-related genes such as cytochrome or membrane transporters has been considered an effective strategy to tailor pharmacological treatments. However, it is widely acknowledged that the patient’s phenotype regarding drug ADME is not only the result of the patients’ genotype but also of a plethora of pharmacological, physiological, pathological, and environmental factors. Although no data are available on DGIs regarding CDKis, many studies have been published concerning the impact of genetic polymorphism in CYPs and transporters for many other drugs. By analogy, it can be speculated that polymorphisms on both cytochromes and transporters involved in CDKis ADME might be relevant as well. Evaluating the still unexplored aspect of DGIs, along with DDIs, in CDKis could be a valuable tool in predicting drug PK variability, especially considering the effect seen in specific population [18]. According to some authors, a different prevalence of *CYP3A4* SNPs between Asian and non-Asian populations could be a possible explanation for the heterogeneity in CDKis efficacy and tolerability profile seen in the recent aggregate data meta-analysis on 2499 patients (*n* = 492 Asians; *n* = 2007 non-Asians) [18,19]. The most relevant SNPs possibly affecting CDKis ADME (*CYP3A4*, *CYP3A5*, *ABCB1*, and *ABCG2*) will be hereinafter reviewed.

### 3.1. Phase I Enzymes: CYP3A4 and CYP3A5

The human CYP3A plays a dominant role in the metabolism of more drugs than any other biotransformation enzyme. CYP3A subfamily is the predominant isoform in both the intestinal epithelium and liver. Genes coding for CYP3A are clustered in the same locus on chromosome 7 and linkage results [64] demonstrated a high degree of sequence homology and substrate overlap specificity between CYP3A4 and CYP3A5. CYP3A expression varies as much as 40-fold in liver and small intestine donor tissues [65]. Increasing evidences have shown that genetic variants in both *CYP3A4* and *CYP3A5* could contribute to the large interindividual variability in CYP3A enzyme expression and activity and ultimately affect response to substrate drugs [66,67]. In the human liver, CYP3A4 accounts for most CYP3A isoform while CYP3A5 is not uniformly present and might be expressed in only 20–30% of people [43]. Over time, few SNPs have shown to affect CYP3A4 expression or activity contrarily to other highly polymorphic CYP3A enzymes (2D6, 2C9 and 2C19).

CYP3A4*22 is an intronic SNP located in intron 6 (*rs35599367*), first reported in 2011 to be responsible of reduced mRNA/protein expression in the C > T variant [68]. Regardless the underlying mechanism is still unknown, CYP3A4*22 is considered so far, the only variant clearly associated with CYP3A4 reduced activity up to 50%. Several studies tested the effect of CYP3A4*22 on PK of drugs. A significant impact of CYP3A4*22 was found on the oncologic tamoxifen [69,70] and sunitinib [71] and not in clopidogrel [72] and tacrolimus [73] metabolism. The relevance of CYP3A4*22 has been hypothesized to be even more enhanced in subjects having no active CYP3A5 enzyme (as most Caucasian individuals). Its allele frequency ranges from 5–8% in Caucasian to 4% in Asian and African population but nonetheless it was shown to be clinically relevant. Regardless CYP3A4*1B (*-392A>G*, *rs2740574*) is the most studied SNP, its functional significance is still considered controversial nowadays and it has been hypothesized that the true cause of the observed clinical phenotype was imputable to the linkage disequilibrium between CYP3A4*1B and another *CYP3A* allele (CYP3A5*1 in Africans and *3 in Caucasians) [67,74]. Other variants have been described in non-exonic regulatory regions, and most of them have no functional significance.

Rare variants may contribute to explain effect on PK observed in the clinic and, especially in drug metabolism, they could provide a promising new approach to explain the observed variation in overall drug response not justified by common SNPs. Novel techniques as next generation sequencing (NGS) are extremely helpful tools in this context. It has been suggested that NGS detection of copy number variations (CNV) of genes involved in ADME of drugs could contribute to further improve genotype-phenotype correlations [75]. Plus, exome sequencing can accelerate pharmacogenetic discovery by assessing both common, i.e., minor allele frequency (MAF) > 5%, and rare (MAF < 1%) variation in virtually all genes in an individual at relatively low cost [76,77].

The only variants identified as causing loss-of-function-mutations and resulting in complete loss of CYP3A4 protein formation were two exonic rare variants: *20 rs67666821 [78] and *26 rs138105638 [79]. Both CYP3A4*20 and *26 caused a premature stop codon yielding a truncated protein devoid of catalytic activity. The first *20, was carried in heterozygosis by a Brazilian woman (calculated MAF in Caucasian population <0.006) while the other, *26, was found in homozygosis in a kidney transplanted patient with Alport syndrome. This latter patients carried also nonfunctional *CYP3A5* (*3/*3 genotype) resulting the first case known to date with complete failure of CYP3A enzyme activity.

No guidelines are published to date on DGIs involving *CYP3A4* nor on how to translate *CYP3A4* genotype to phenotype. However, based on the current evidences, *CYP3A4* poor metabolizer (PM) status can be defined by the presence of aforementioned loss of function rare variants. Individuals with one copy of the CYP3A4*22 allele will have the predicted phenotype of normal/intermediate metabolizers (NMs/IMs, respectively) while individuals with two variant copies can be considered IMs. Individuals without any *22 variant will therefore be NMs.

CYP3A5*3 (*rs776746*) is the most extensively studied *CYP3A5* SNP and it confers a variant in intron 3, leading to aberrant splicing and nonfunctional CYP3A5. Its allele frequency varies from approximately 50% in African-Americans to 90% in Caucasians. Additional and less frequent variations as CYP3A5 *6 (mainly in African 15–25%) and *7 (Asians 1%) have been associated with decreased CYP3A5 activity, similar to the one observed with *3. Several other *CYP3A5* variants have been described but occur at relatively low allelic frequencies and their functional significance has not been established yet. The CYP3A5*1 allele produces the functional protein (defining the expresser phenotype and normal metabolizer, NM), whereas the common CYP3A5*3 allele together with *6 and *7 will identify the nonexpresser phenotype. Specifically, an individual carrying one functional allele and one nonfunctional allele are IMs while an individual carrying two nonfunctional alleles will be PMs. Guidelines have been published by CPIC regarding tacrolimus dose-adjustment based on the evidence that individuals with the expresser phenotype may require higher dose compared with nonexpressers [80].

### 3.2. Phase II Enzymes: SULTs

Sulfate conjugation catalyzed by cytosolic SULTs enzymes is an important pathway in the biotransformation of many drugs, neurotransmitters, and steroid hormones. SULTs enzymes display a wide interindividual variability, even though its clinical impact on drug adverse effects and efficacy has not been elucidated yet [63]. Such variability was only partially explained by genetic variation, suggesting that other non-genetic, epigenetic, and environmental influences could be major determinants of variability in SULT activity [62].

SULT2A1 is a member of this family of enzymes and is highly expressed in the human liver, adrenal cortex, and lastly small intestine [62]. Among endogenous compounds are listed steroids (androsterone, allopregnanolone, dehydroepiandrosterone) and bile acids.

Ethnic-specific pharmacogenetic variants in *SULT2A1* might influence the biotransformation of both orally administered agents and/or endogenous substrates. SNPs and CNVs have been proposed as a source of genetic variability in SULT2A1 encoding gene. Two *SULT2A1* SNPs (*rs2637125* and *rs182420*) altering the amino acid sequence [81] and one CNV resulting in a deletion/insertion of a non-coding 2849bp [82] have been identified and associated with altered SULT2A1 activity. These variants may contribute to individual variation in response to drugs, however current knowledge on SULT interindividual variability is based on in vitro/in vivo pharmacogenetic and expression/activity study. Studies in humans are lacking [63].

### 3.3. Impact of Genes with Indirect Impact on CYP3A Activity

Variations in the *CYP3A4/5* genotype contribute only to a minor extent to the interindividual differences while a major cause of variability could derive from NRs, cytokines or competitive inhibition of the CYP3A-mediated drug metabolism. Variants in genes as *NR1/2*, *PPARα* and P450 oxidoreductase (*POR*) have been lately identified to have an indirect impact on CYP3A activity. Variants also in *PXR* and vitamin D receptor (*VDR)* could play a role in CYP3A4 induction [61,83,84]. A few clinical studies have reported higher tacrolimus dose requirement, indicating elevated metabolic activity of the CYP3A, in association with *NR1/2 rs2276707* and with POR*28 rs1057868 [85]. In this latter case the noted effect was only in patients CYP3A5 expresser. While CYP3A4 mRNA expression was significantly reduced in cells derived by patients’ homozygous carriers of *PPARα rs4253728* [67].

Members of the NR family (farnesoid X receptor, FXR; CAR; PXR and ERα) are important regulator of SULT2A1 transcription as well.

### 3.4. Transporters (ABCB1 and ABCG2)

Despite evidence for interindividual variability in ABCB1 expression and function, so far, all *ABCB1* described SNPs have not shown consistent association neither with drug PK nor PD. The three most studied variants in *ABCB1* are two synonymous SNPs, *1236C > T*, (*rs1128503*) in exon 12 and *3435C > T* (*rs1045642*) in exon 26; and one non-synonymous SNP, *2677G > T/A* (*rs2032582* in exon 21). These SNPs are in high LD [86] defining ABCB1*13 haplotype and have been observed in most ethnic groups. Considering the controversial results obtained so far, no adjustment in drug dosing have been recommended for individuals carrying sequence variants of *ABCB1* and further studies are warranted to draw any conclusion. However, considering that the presence of these variants has been associated with a lower P-gp expression [87] it could identify an ineffective efflux pump functionality phenotype with the consequence on bioavailability of orally administered CDKis.

As per P-gp also BCRP, encoded by *ABCG2* gene, is an efflux transporter responsible of limiting oral drug bioavailability of many common medication and transport across tissue-brain barriers. It is localized in the gastrointestinal tract, liver, kidney, mammary tissue, placenta, testes, and brain endothelium. SNPs on *ABCG2* have demonstrated to be closely related to interindividual variability in therapeutic response. The two most common missense variants are *c.34G>A* (*rs2231137*) and *c.421C>A* (*rs2231142*). According to several studies, the *c.34G>A* variant has no appreciable effect on the expression or function of the transporter while *c.421C>A* was shown to reduce expression of BCRP and result in lower efflux of substrates. Both SNPs have a higher allele frequency in East Asians (30–60%) compared to Caucasians and African-American (5–10%). *ABCG2 c.421C>A* genotype was found to be a significant determinant of imatinib [88], gefitinib [89] and sunitinib [90] PK in cancer patients. Regarding erlotinib, the same association, with consequence on toxicity risk, was found to be significant only in Asian patients [91,92].

## 4. Phenoconversion

According to some authors, the reason of the conflicting results often observed in DGIs association studies, lies in the lack of consideration of DDIs and/or other pathophysiological features that impact drug metabolizing enzyme activities. Phenoconversion is a phenomenon that occurs when genotypic NMs are converted into phenotypic IMs or even PMs of drugs, with a modification of their clinical response to that of genotypic PMs [25]. While genotype-predicted PMs (based on the detection of two no-function or severely reduced-function alleles) reliably predicts a PM phenotype, the same cannot be claimed for individuals with genotypes predicting NMs/IMs phenotype. Pharmacogenetic studies do not focus on the prevalence of genotype–phenotype discordance in non-PM subjects [25]. This is an issue for the appropriateness of genotype-based prescribing decisions in non-PM subjects. Two main factors of phenoconversion involves co-medications that inhibit or alter the activity of a drug metabolizing enzymes, or pathophysiological features associated with co-morbidities that impact expression levels of drug metabolizing enzymes [25]. In cancer treatment phenoconversion, due both to genetic polymorphisms and clinical alterations induced by tumor itself, plays a key-role in the efficacy of therapies. Phenoconversion has the potential to influence pharmacological properties of employed drugs in terms of PK parameters, especially AUC and C_max_, concurring to the interindividual variability often seen among healthy subjects and patients [25,43,93].

An example of phenoconversion induced by DDIs consists in the potent CYP2D6 inhibitor paroxetine that is often prescribed with the CYP2D6-activated tamoxifen as tamoxifen-induced hot flushes treatment along with depression treatment. According to some authors, *CYP2D6* PM genotype could not be significantly associated with an increased risk of tamoxifen failure because of the lack of concern toward CYP2D6-related DDIs. Such interactions could help identify a higher number of *CYP2D6* PMs phenoconverted from NM, IM, or even ultra-rapid metabolizers (UMs) status. According to a population-based cohort study published last year, paroxetine use during tamoxifen has been associated with an increased risk of death from breast cancer directly related to the extent of co-prescribing, supporting the hypothesis that paroxetine can reduce or abolish the benefit of tamoxifen in women with breast cancer [94]. When 75% of the time on tamoxifen was overlapped with paroxetine, the adjusted hazard ratio (adjHR) for survival was 1.91, when 50%, the adjHR was 1.54, while when 25%, the adjHR was 1.24.

## 5. Potential Drug-Pathophysiological Interactions

Many clinical conditions as cancer and cardiovascular diseases are known to have inflammatory components, potentially influencing the activity of drug metabolizing enzymes. Besides inflammation, other clinical conditions (e.g., hepatic or renal impairment) or pathophysiological conditions as gender, women’s hormonal status, obesity and age have been hypothesized to modify cytochromes activities.

### 5.1. Hepatic Impairment

Hepatic impairment can affect drug disposition and metabolism according to the underlying liver disease and its severity. Severe hepatic diseases, up to cirrhosis, can reduce hepatic clearance, metabolism and drug bioavailability through different mechanisms such as: (i) the capillarization of the sinusoids, consisting in the occlusion of the fenestration of sinusoidal endothelium which limits the drug uptake from the hepatocyte [95,96]; (ii) the overall inadequate hepatic enzymatic activities; (iii) the reduced production of plasma proteins.

For patients with mild to moderate hepatic disease, no dose adjustment is required, while in the case of palbociclib, patients with severe hepatic impairment (Child-Pugh class C) must be treated at the dose of 75 mg once daily [35,45].

For ribociclib, a PK study in patients with hepatic impairments has been conducted, showing no effect in the exposure of the drug in patients with mild impairment while in those with moderate or severe impairment, exposure was increased less than two-fold. The results were confirmed also in another analysis comparing 160 patients with breast cancer and normal liver function with 47 with mild liver impairment.

Regarding abemaciclib, a PK study in patients with different degrees of liver impairment has suggested a similar exposure of the drug in those with mild or moderate liver injury, though a lower C_max_ has also been described. In severe liver injury, exposure was 2.09 (90% CI: 1.33, 3.28) times higher with respect to patients with normal liver function. Similarly, half-life and T_max_ were longer. In contrast, the AUC of abemaciclib active metabolites was decreased. Globally, the increase in parental drug exposure was outweighed by the decrease in metabolites exposure. As already described in the PK Section 1.3, abemaciclib is highly bound to plasma protein. Its plasma protein binding decreases with increasing severity of hepatic impairment. The average unbound fraction is 3.7% in patients with normal liver function and increases up to 5% and 7.8% in those with moderate and severe impairment respectively [16,46].

Although the overall reported injury of hepatotoxicity in CDKis in clinical trials has been less than 10%, palbociclib has been associated with liver injury in some case reports [97,98,99]. In three of the four reported cases, it was described a fatal liver injury.

Phase III trials with ribociclib have registered a relatively high incidence of hepatobiliary toxicity, mainly grade 3 and 4 toxicities [7,10,11,13].

**Figure 2:**
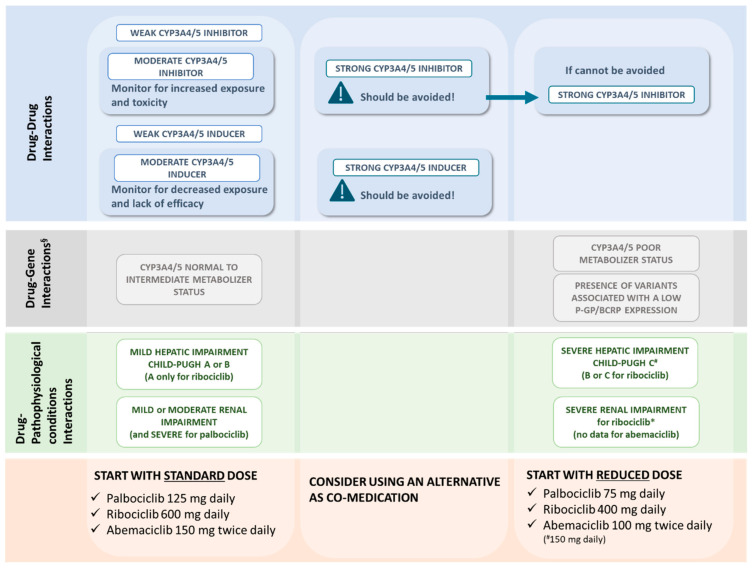
Starting dose selection of palbociclib, ribociclib and abemaciclib, according to DDIs, DGIs or interactions with pathophysiological conditions. No studies have been performed in hemodialyzed patients for all the three CDKis. * In patients with severe renal impairment, treated with ribociclib, the starting dose which is approved by EMA is 400 mg/daily vs. 200 mg/daily by FDA. # The suggested reduced starting dose for abemaciclib is 150 mg daily. **§** No evidences available and no dosing is suggested.

Details on dose adjustments related to hepatic impairment are reported in Table 4 and Figure 2.

### 5.2. Renal Impairment

In a PK dedicated trial, patients with different degree of renal impairment, excluding hemodialyzed, received palbociclib and no dose adjustment was required in any case [NCT02085538]. These data are consistent with a population PK analysis including 73 and 29 patients with mild renal and moderate renal impairment respectively, who did not show modified exposure to palbociclib [35,45].

In a population treated with ribociclib, PK analysis comparing 77 patients with normal renal function, with 76 patients with mild renal impairment and 35 with moderate renal impairment, no differences in the exposure to the drug have been noticed among the three groups. Recently a phase I study, aiming to evaluate ribociclib PK and safety profile in patients with varying degrees of impaired renal function compared to healthy volunteers with normal renal function, confirmed that no dose adjustment is necessary in patients with mild to moderate renal impairment. A starting dose of 400 mg once daily is recommended in patients with severe renal impairment [NCT02431481] [100]. Nevertheless, the FDA suggests an even further reduced dose of 200 mg once daily [10].

Abemaciclib has no dedicated PK studies in patients with renal impairment. As described in Section 1.3, their metabolism is predominantly hepatic, and thus, according to preclinical studies and population PK analyses, no modified excretion is expected in mild to moderate renal impairment and no dose adjustment is needed [101].

Details on dose adjustments related to renal impairment are reported in Table 4 and Figure 2.

### 5.3. Gender and Hormonal Status

Researchers have shown how gender differences may contribute to variation in safety and efficacy of drug therapy. One of the main explanations of such difference lies in PK and pharmacogenetic differences. In fact, hormones can modify transporters and enzymes expression as well as play a role in gastric emptying. Progesterone and estrogen can modulate gastric pH and time of transit in stomach and bowel [102]. Woman in fact are characterized by a higher gastric pH and a lower bowel transit [102]. As previously discussed, palbociclib absorption is highly dependent on gastric pH, also abemaciclib absorption is influenced by food intake and this may be due to pH modifications. On this base, we can expect a lower exposure in women with respect to men. Ribociclib absorption, instead, is not affected by food intake [103]. Furthermore, bioavailability changes in association with gastric pH must be considered even more because of the frequent association of oncological therapies with gastric pH elevating drugs, such as proton pump inhibitors or H2 blocking agents.

Differences between males and females which can impact in drug exposure, have been described also in association with transporters and drug metabolizing enzymes. At the intestine level, no differences in P-gp expression between male and female have been described [104,105] but in liver, P-gp expression is twice as high in females compared to male and half as much for CYP3A4 activity [106]. Some studies have also demonstrated a higher liver expression of BRCP in males with respect to females [107]. This can be translated in a higher clearance rate of palbociclib and abemaciclib in males, potentially offsetting metabolism differences for these drugs. Also, for drug-induced QT prolongation, significant differences exist between male and female. In fact, women have higher risk to incur in TdP because of QT prolongation [108]. Rodriguez et al. have also demonstrated an association between hormonal changes during menstrual cycle and QT lengthen [109]. Thus, we expect a higher risk of QT prolongation for women, especially those in premenopausal, that should be taken into consideration when the CDKi of choice is ribociclib. Indeed, it has been proposed that natural fluctuation in endogenous sex steroid hormones with the menstrual cycle, pregnancy, and menopause could potentially influence drug efficacy and safety profile overall [110]. The physiologic decrease in circulating estrogen in menopause has been associated with changes in drug metabolizing enzymes. According to Paine et al. CYP3A4 activity in the intestine was 20% lower in the post-menopausal compared to pre-menopausal women [104]. The effect of menopausal status on the PK of drugs has been poorly investigated. The pre-menopausal status has been associated with decreased endoxifen plasma concentrations by 135% compared to post-menopausal status in tamoxifen-treated breast cancer patients phenotyped as NMs for CYP2D6 and with CYP3A activity [111]. The authors hypothesized that a lower P-gp activity in the gut of post-menopausal patients could be held responsible for the increased tamoxifen bioavailability. It may be conjectured that substrate drugs for CYP3A4 and P-gp, as CDKis, may increase their plasmatic concentrations and toxicity in the post-menopausal status.

Finally, it is worth reminding that CDK 4/6 inhibitors have been approved only for breast cancer in women. Although this pathology is an almost exclusively female condition with less than 1% of all breast cancers occur in men [112], further studies on CDKis and male breast cancers are needed in order to provide supporting evidences for CDKis in this clinical setting. Furthermore, due to the relevant pathophysiological involvement of sexual hormones in breast cancer, several preclinical and clinical studies are ongoing to test CDKis in other endocrinological tumors such as ovarian cancer [113].

### 5.4. Inflammation and Cancer

Inflammation, caused by infectious disease or other conditions like cancer, has been associated with the downregulation of several drug metabolizing enzymes expression [114,115] and, as previously mentioned, can drive the phenoconversion phenomenon [25]. Cytokines such as IL1, TNF, interferons and IL-6 are primarily involved in this mechanism [116]. Proinflammatory cytokines can suppress biosynthesis of drug metabolizing enzymes impacting their quantities, thus affecting their capacity of drug metabolization. This influence has been clearly demonstrated in in vitro [117,118,119] and in vivo [120,121,122] experiments. In confirmation, Rivory et al. demonstrated that, in advanced cancer patients, the ones with acute phase response (CRP > 10 mg/L) presented a 30% lower CYP3A metabolic activity with respect to patients without acute phase response. This observation did not take account of the type of oncological disease, nevertheless 9% of studied patients were suffering from breast cancer [122]. Such phenoconversion can alter drug metabolization, exposing patients to a higher risk of adverse events from cancer therapy. Neoplastic tissue can also contain cytochrome P450 enzymes with the capacity to metabolize drugs. A study on breast carcinomas has revealed the overexpression in cancer tissues of CYP3A4 and CYP2C9. Another study from 2016 hypothesized that the PXR-mediated induction of CYP3A5 expression, common in a range of solid tumors, could increase metabolism of taxanes and tyrosine kinase inhibitors [123]. The effects of the expression of these enzymes are still unexplored, but they have been proposed to play a role as a resistance mechanism [124]. A better-known mechanism of resistance consists in the increased activity or synthesis of ABC efflux transporters by the tumor, facilitating the efflux of cancer drug and reducing intracellular drug concentrations. Elevated levels of P-gp have been found in more than half of the NCI-60 tumor cell lines [125].

Probably, NRs activity could be modified in flogosis, since they are considered important xenosensors mediating the impact of inflammation on the expression of ADME genes and finally on therapy outcome [84].

With regard to phase II drug metabolizing enzymes, a reduction in sulfonation capacity has been linked with the acute-phase inflammatory response of several infectious and inflammatory conditions. An altered drug metabolism and the development of cholestasis have been proposed to be associated with SULT2A1 suppression in inflammation. Sulfotransferase activity decreases significantly with the severity of liver disease from steatosis to cirrhosis, potentially causing an increase in drug levels and toxicity [63,126].

### 5.5. Brain Metastases from Breast Cancer

Brain metastases occur in 10–16% of patients with breast cancer [127]. Unfortunately, registration studies have excluded patients with brain metastases or when they have included them, as in the MONALEESA-3 case, specific outcomes on CNS were not discussed. In a condition of inflammation like that caused by brain metastases, drugs penetrability through the BBB could potentially be increased. BBB is responsible for ensuring the homeostasis of the CNS and this function is explicated by the CNS endothelial cells which are rich of tight junctions predominately belonging to the family of claudin (especially Cldn5 and Cldn3) and occludin. Also, pericytes, astrocytes, and microglial cells are responsible for the impermeability of the BBB. Inflammation is linked with BBB disruption and leakage, and this is associated with the reduced expression of claudins and occludins caused by inflammatory mediators (IL-1β, IL-6, IL-17, IFN-γ, TNF-α). These cytokines are also able to up-regulate chemokines and cell adhesion molecules expression thus modifying BBB permeability [128].

Penetrability across the BBB could be further increased considering that P-gp seems to be not so extensively expressed in the BBB of patients with metastatic brain tumor [129] and that all CDKis are substrates of P-gp-mediated extrusion from the brain. An experimental model comparing the BBB permeability of palbociclib and abemaciclib, more lipophilic than ribociclib, has shown that the efflux efficiency is lower for abemaciclib than palbociclib. In vitro efflux ratios were respectively of 4.1 and 12, respectively [51]. Nonetheless, used doses are much higher than clinically relevant doses. A phase I trial has shown that concentrations of abemaciclib can be measured in cerebrospinal fluid of patients and radioactivity from 14C marked abemaciclib can be measured after a single oral dose of 10 mg/kg up to 12 h [130]. An intracranial clinical benefit for 58 patients with brain metastases secondary to HR+ metastatic breast cancer treated with abemaciclib have been demonstrated by Anders et al. at the ASCO 2019 [131]. Specifically, 6% of patients had confirmed objective intracranial response and 38% a decrease of intracranial lesions. Intracranial clinical benefit rate persisting for ≥6 months was 25%, while median PFS was 4.4 months (95% CI, 2.6–5.5) [131].

A good CNS penetration has also been shown for ribociclib in mice bearing glioma cortical allograft tumors vs. non-tumor bearing mice [52], as well as in a PK analysis of a phase 0 trial involving recurrent glioblastoma patients, however with inconclusive results in terms of clinical efficacy [132] that require further investigations.

In the peculiar conditions of brain metastases, palbociclib could potentially reach therapeutic concentrations as well, regardless being a major P-gp and BCRP substrate at the BBB. Indeed, results of an interim analysis of a phase II trial of palbociclib administration in patients with brain metastases harboring CDK pathway alterations [NCT02896335] presented at the 24th Annual Meeting and Education Day of the Society of NeuroOncology, revealed that 57% of the patients evaluable were having an intracranial response after treatment with palbociclib with a median overall survival of 6.5 months (90% CI, 3.8–13.6) [133].

### 5.6. Obesity

Obese patients are characterized by a marked modification in drug distribution and elimination. These patients are typically characterized by a higher first pass metabolism caused by an increase in hepatic blood flow and hepatic clearance. Furthermore, a higher prevalence of obese patients suffers from a condition known as NASH (non-alcoholic steato-hepatitis), characterized by fatty infiltrations and liver inflammation. The 90% of liver biopsies of obese subjects have shown to have these characteristics [134]. Incidence of NASH increases with Body Mass Index (BMI) and its prevalence is estimated to be up to 20% of the obese population, rising to 50% in morbidly obese patients. As previously discussed, inflammation can cause a decrease in drug metabolizing enzymes expression.

Moreover, renal clearance is affected by obesity, in fact these patients are associated with a state of glomerular hyperfiltration resulting in a condition of enhanced renal clearance.

Since several studies have shown a significantly stronger association between increased BMI and higher breast cancer incidence. This is especially true for postmenopausal women, where several meta-analyses have consistently shown positive associations among high adiposity, adult weight gain and risk of HR-positive breast cancer [135,136,137]. Moreover, a study revealed that 47.7% of patients diagnosed with breast cancer were obese [138]. Because of the very high prevalence of obese patients in this population, further studies to characterize PK in obese patients are needed, as recommended by the investigation of PK and PD in the obese population published by the EMA-CHMP [139].

### 5.7. Age

Several modifications in drugs PK are induced also by age. Starting from drug absorption, elders have reduced gastric acid secretion [140,141], exposing patients to a higher absorption of CDKis. Furthermore, concurring to a higher exposure, elders are characterized by a reduction in first-pass metabolism [142,143], probably because of a reduction in the hepatic blood flow and in tissue mass. Significant changes in body mass composition and fat distribution, altering a lot the distribution of drugs in tissues have been associated with ageing process. Palbociclib and ribociclib are characterized by a high volume of distribution and because of the age-related modifications, elders can result in higher serum concentrations. Despite there are no evidences of alterations in the expression of serum proteins with the age, prevalence of malnutrition among elderly is high [144] and this can have an impact in serum albumin concentrations, which can result in a higher free fraction of CDKis. In women, contrary to men, age induces also a reduction in the expression of P-gp [145], affecting once again drug bioavailability. All these considerations warrant particular caution in the treatment of elderly patients, considering that breast cancer risk increases with age and more than one third of patients diagnosed with breast cancer are over 70 years of age [146]. Since it is a population of fragile patients at an increased likelihood of adverse events both because their oncological disease and their age, the use and dose of CDKis should be the most appropriate to avoid exposing them to excessive concentrations of these drugs.

## 6. Mechanisms of Resistance to CDKIs

The improvements of clinical outcomes obtained with CDKis are jeopardized by the occurrence of resistance to these treatments in some patients [147]. This phenomenon can be due to intrinsic and/or acquires mechanisms which can be classified as cell cycle-specific and non cycle-specific [148]. Taking in consideration the molecular pathways CDKis target, the most relevant causes of cell cycle specific resistance are all that conditions which promote the G1–S transition of the cell cycle, such as: (i) the loss of tumor suppressor proteins as RB and/or FZR1 [149]; (ii) amplification of proteins involved in the CDK-complex as CDK2, CDK4, CDK6, p16, CCNE1/2, and E2F [148]; (iii) overexpression of CDK7 which support CDK4/6 activity [150]; (iv) activation of HDAC which inhibits CDK suppressors [151]; (v) alteration of other molecular check-points of cell-cycle as the overexpression of WEE1 [148]; (vi) overexpression of proteins which inhibit cellular senescence as MDM2 [152]. On the other hand, every other non-specific pro-oncogenic alteration not involving cell cycle specific pathways can induce resistance, such as: (i) the activation of the FGFR and PI3K/AKT/mTOR pathways; (ii) the loss of ER or PR expression; (iii) the higher transcriptional activity of AP-1 and/or EMT pathway; (iv) the suppression of SMAD3 pathway; (v) the autophagy activation; (vi) immune mechanisms [148,153].

To date, several studies aim to identify patient profiles or biomarkers in order to predict the resistance to CDKis, but no strong evidences yet emerged, and further studies are required for this relevant unmet medical need [153,154].

## 7. Discussion

Current clinical questions include how to choose among palbociclib, ribociclib, and abemaciclib and to personalize their use according to the need of each particular clinical setting in breast cancer [155]. The factors concurring in defining patient’s metabolizer phenotype and consequently the inter and intra-patient variability associated with CDKis use are diverse. Indeed, DDIs, DGIs and patient’s pathophysiological conditions can modify CDKis pharmacological features amplifying differences in CDKis safety and efficacy profile. Drugs alter metabolizing enzymes, transporters, and/or efflux pumps, inducing PK modifications that influence their efficacy or the efficacy of other co-administered compounds. Consequently, therapeutic concentrations as well as their cytotoxic effects could therefore undergo changes.

Germline host characteristics have been considered so far responsible for a major proportion of the observed interindividual variability in many drugs. Subpopulation analyses in breast cancer patients treated with CDKis highlighted differences in their safety as well efficacy profile. Different prevalence in *CYP3A4/5* and transporters (*ABCB1* and *ABCG2*) SNPs among subpopulations have been proposed to play a role in influencing ADME of CDKis PK. At the same time, some pathophysiological conditions can cause a variability in PK parameters, both intra-patient as food intake, or interpatient as renal or hepatic impairment, systemic or local inflammation status, general clinical condition, hormonal profiles, and eventually tumor grading/staging.

**Figure 3:**
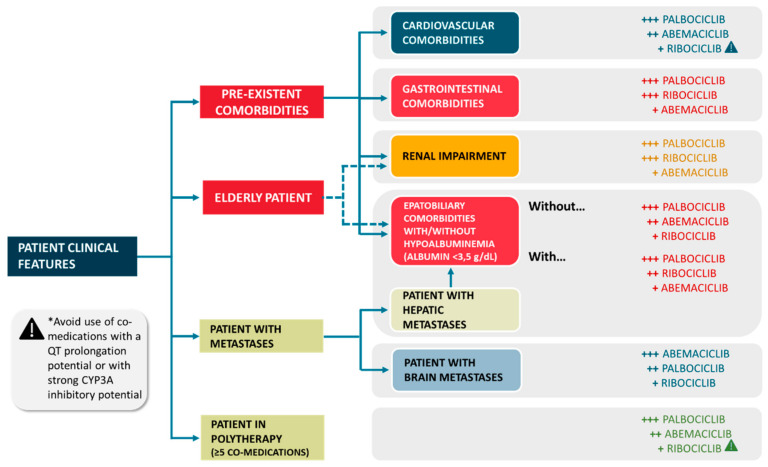
Flow diagram for potential CDKi selection to increase individual safety and tolerability, according to safety data from literature. The CDKi choice of priority is based on each patient’s clinical feature and is arbitrary expressed by: +++, highly recommended to use; ++, moderately recommended to use; +, lower recommended to use.

Given the pharmacological considerations about each CDKi, we can speculate that some clinical settings could be more suitable than others for the adoption of a specific CDKi. A tailored therapeutic approach taking into consideration all the factors potentially contributing to an altered PK/PD profile (based on DDIs, DGIs and pathophysiological condition), could represent indeed an innovative safer solution. It could also represent an effective strategy in the clinical decision making of CDKis use (Figure 2 and Figure 3).

For example, abemaciclib should be avoided in patients with pre-existent gastrointestinal comorbidities since severe nausea, vomiting and diarrhea are the most frequent adverse events which are also able to alter abemaciclib PK profile. On the contrary, it is worth to underline that abemaciclib has been associated with the safest toxicological profile among CDKis, due to the less frequent neutropenia events. For patients with cardiovascular comorbidities palbociclib should be preferred seeing the high risk of thromboembolic events [156,157] with abemaciclib and QT prolongation risk associated with ribociclib. This is clinically relevant in breast cancer settings, considering also that women are at higher risk of QT prolongation, since their QT interval is usually longer compared to man [50]. In patients with mild hepatic impairment, ribociclib should be avoided since it may induce hepatotoxicity more frequently compared with the other CDK4/6 inhibitors.

Palbociclib and ribociclib are characterized by lower binding to serum protein and thus should be preferred in patients with advanced metastatic disease and hepatic localization, considering that hepatic dysfunction can lead to hypoalbuminemia and consequently to a larger amount of unbound (free) drug. Hypoalbuminemia may induce toxicity in patients treated with abemaciclib, highly bound to serum protein. In renal impaired patients palbociclib seems to be the safest choice according to results deriving from trials in this specific population whereas less evidences are available for the other CDKis. Similarly, palbociclib could be preferred in patients in polytherapy (defined as more than five co-medications) for comorbid conditions due to a lower incidence of clinically meaningful DDIs. These suggestions should be more accurately taken in consideration in elderly patients where hypoalbuminemia, renal impairment and polytherapy may more frequently coexist (Figure 3).

Abemaciclib could be hypothesized to be the drug of choice in patients with brain metastases, because of its lipophilicity along with its ability to inhibit P-gp efflux pumps and to reach therapeutic concentration in brain tissue with lower doses compared to the other CDKis, in addition to its multiple mechanisms of action involving CDK1 and CDK2, which are implicated in brain oncogenesis [158].

In conclusion, many pharmacological variables should be considered when choosing one treatment or another. CDKis are not only subjected to the well-known variability related to the oral administration route, but also to the less studied one deriving from interacting comedications, pharmacogenetic profile and pathophysiological conditions in cancer patients. The use of a specific CDKi should shift from an empirical approach to a more personalized one aimed both at reducing sources of variability and at tailoring dose to the individual patient, also through the help of therapeutic drug monitoring. Dose individualization is one of the principal challenges facing personalized medicine in such a complex setting as cancer treatment, especially in patients affected by this big killer.

All these considerations could have also other relevant implications because CDKis have been studying for other clinical settings. Indeed, their efficacy is under investigation in HER2-overexpressing and triple-negative breast cancer [159], although palbociclib as a monotherapy has already shown no efficacy in triple-negative breast cancer [160]. Furthermore, due to their complex and not deeply understood mechanisms of action, CDKis, especially abemaciclib, have shown as monotherapy encouraging results in preclinical models of other tumor such as glioblastoma, non-small cell lung cancer, head and neck squamous cell carcinoma, pancreatic cancer, esophageal adenocarcinoma, melanoma, colon cancer, myeloma, and ovarian cancer [113,161,162]. Analogously, since CDKis influence a wide range of key functional proteins, several studies have investigated possible therapeutic strategies combining CDKis to drugs targeting immune checkpoint such as PD-1/PD-L1 [163,164], or other molecules interacting with the PI3K-AKT-mTOR pathway [165,166].

To conclude, CDKis are promising therapeutic agents which need to be more deeply studied in all their clinical pharmacological properties in order to be appropriately exploited to represent the best safety and efficacy treatment option.

## Figures and Tables

**Table 1:** Molecular pathways of kinases which are targeted by palbociclib, ribociclib and abemaciclib. The grading of affinity between CDKis and each target kinase is arbitrary expressed by -, no affinity; +, presence of affinity; ++, high affinity; +++, very high affinity.

Targeted Kinase	Pathophysiological Activities of Targeted Kinases	Affinity of Palbociclib	Affinity of Ribociclib	Affinity of Abemaciclib
CDK1	It is mainly involved in controlling the transition from G2 to M phase of cell-cycle	-	-	+
CDK2	It selectively orchestrates processes of phase S, binding Cyclin E, and not Cyclin D as for the other CDKs	-	-	+
CDK4	It inhibits members of the retinoblastoma (RB) protein family including RB1 and regulate the cell-cycle during G(1)/S transition [34]	++	+++	+++
CDK6	It inhibits members of the retinoblastoma (RB) protein family including RB1 and regulate the cell-cycle during G(1)/S transition [34]	++	++	+
CDK7	It regulates the initiation of transcription through phosphorylation of the heptad repeats that comprise the C-terminal tail of RNA polymerase II (CTD)	-	-	+
CDK9	It regulates the release from promoter proximal arrest of transcription through phosphorylation of the heptad repeats that comprise the C-terminal tail of RNA polymerase II (CTD)	-	-	++
GSK3 α/β	It promotes the synthesis of pro-inflammatory IL-6 and the expression of oncogenic genes	-	-	+
CAMKII α/β/γ	It is involved in apoptosis and autophagy in cancer cells	-	-	+
DYRK	It regulates some proteins controlling the cell cycle	-	-	+
PIM protein kinase	It is an oncogenic protein which is frequently amplified in cancer	-	-	+
HIPK	It promotes JAK/STAT signaling	-	-	+
CAMK families	They are enzymes overexpressed in several cancer types	-	-	+

CDK—Cyclin Dependent Kinase; GSK3 α/β—glycogen synthase kinase 3α/β; CAMKII α/β/γ—calmodulin-dependent protein kinase II α/β/γ; DYRK—dual-specificity tyrosine phosphorylation-regulated kinase; HIPK—homeodomain-interacting protein kinase; CAMK—Ca^2+^/calmodulin-stimulated protein kinase; JAK—Janus Kinase; STAT—Signal Transducer and Activator of Transcription.

**Table 2:** Summary of more relevant adverse events (grade 3 or 4 according to NCI-CTCAE) in registration trials of palbociclib, ribociclib and abemaciclib. CDKi, CDK4/6 inhibitor; ET, endocrine therapy; AI, aromatase inhibitors.

CDKi	Registration Trial	ET Backbone	Patients Reporting Adverse Events with Grade 3 or 4 of (%)							
			Neutropenia	Leukopenia	Anemia	Infections	Nausea	Vomiting	Diarrhea	Fatigue
Palbociclib	PALOMA-2	AI	66.5	24.8	5.4	0	0.2	0.5	1.4	1.8
	PALOMA-3	Fulvestrant	76.1	33.8	2.8	3.2	0	0	0	0
Ribociclib	MONALEESA-2	Letrozole	59.3	21	1.2	4.2	2.4	3.6	1	2.4
	MONALEESA-3	Fulvestrant	57.1	15.5	3.9	7.7	1.4	14.1	0.6	1.7
Abemaciclib	MONARCH-1 (monotherapy)	-	28.9	27.7	0	-	4.5	1.5	19.7	12.9
	MONARCH-2	Fulvestrant	26.5	7.3	5.8	4.9	0.9	1.2	13.4	1.8
	MONARCH-3	AI	23.8	8.6	7	-	1.2	1.5	9.5	1.8

**Table 3:** Summary of pharmacological properties of palbociclib, ribociclib and abemaciclib in detail.

Pharmacological Features		Palbociclib [45]	Ribociclib [10]	Abemaciclib [46]
Dosage and schedule		125 mg/daily day 1–21 Q28 with food	600 mg/daily day 1–21 Q28	200 mg twice daily in monotherapy; 150 mg twice daily in combination with endocrine therapy
Selectivity		CDK4 = CDK6 [27]	CDK4 > CDK6 [28]	CDK4 >> CDK6; low potency to CDK1, CDK7 and CDK9 [31]
Lipophilicity; BBB penetration		cLogP value of 5,5; + [47]	N.A.	cLogP value of 2.7; +++ [47]
PK		C_max_: 52 ng/mL T_max_: 7 h t_1/2_: 25.9 h Vd: 2793 L AUC 0–10 (ng/mlxh): 299 [48]	C_max_: 1000 ng/mL (higher value for Asiatic people) T_max_: 5 h t_1/2_:32.6 h Vd: 1090 L AUC 0–24 (ng/mlxh): 20000 [24,49]	C_max_: 298 ng/mL T_max_: 8 h t_1/2_: 8 h Vd: 690.3 L AUC 0–24 (ng/mlxh): 5520
Bioavailability		46%	N.A.	45%
Binding protein		85%	70%	96–98%
Metabolism		Hepatic: substrate of CYP3A and SULT2A1 [48]	Hepatic: substrate of CYP3A4	Hepatic: substrate of CYP3A4
Excretion	In feces	74%	69.1%	81%
	In urine	17%	22.6%	3.4%
Effect on ADME enzymes + autoinhibition		Weak and time-dependent inhibitor of CYP3A. Palbociclib is a substrate of P-gp and BCRP and inhibits OCT1. [7]	Moderate/strong dose- and time-dependent inhibitor of CYP3A4. Ribociclib is a substrate of P-gp. Reversible CYP1A2, 2E1 inhibitor. Potentially inhibits P-gp, BCRP, OATP1B1, OATP1B3, OCT1, OCT2, BSEP and MATE1.	Abemaciclib is a substrate of P-gp and BCRP and inhibits OCT2 and MATE
Active metabolites		No [40]	No	Yes: *N*-desethylabemaciclib (M2), hydroxyabemaciclib (M20), hydroxy-*N*-desethylabemaciclib (M18)
Food intake alteration		Absorption and drug exposure lower in fasted state [40]	No	High fat and high caloric meal increase AUC (9%) and C_max_ (26%)
Adverse events		Neutropenia G3/4	Nausea any grade	Diarrhea any grade; Fatigue any grade. Neutropenia (rare and manageable)
Effect of co-administered CYP3A inhibitors		↑87% AUC ↑34% C_max_	Strong inhibitors: ↑3.2-fold AUC and ↑1.7-fold C_max_; Moderate inhibitors: ↑1.9-fold AUC and ↑1.3-fold C_max_ (after a single 400 mg dose)	Strong inhibitors: ↑237% AUC (↑119% of the active metabolites) and ↑30% C_max_ (↑7% of the active metabolites)Moderate inhibitors: ↑1.7-fold AUC (↑1,3-fold of the active metabolites)
Effect of co-administered CYP3A inducers		Strong inducers: ↓85% AUC and ↓70% C_max_; Moderate inducers: ↓32% AUC and ↓11% C_max_ [7]	Strong inducers: ↓89% AUC ↓81% C_max_ Moderate inducers: ↓60%AUC ↓37% C_max_ (after a single 600 mg dose)	Strong inducers: ↓67% AUC of parent drug and active metabolites Moderate inducers: not known
Pediatric use		No data	No data	No data
Geriatric use		No differences on safety and efficacy	No differences on safety and efficacy	No differences on safety and efficacy

PK—pharmacokinetics; CDKs—Cyclin-dependent kinases; AUC—Area Under the Curve; C_max_—the highest concentration in blood; T_max_—the time a drug takes to reach its peak in blood concentration; t_1/2_—drug half-life; Vd—Volume of Distribution; cLogP value—logarithm of a drug partition coefficient between n-octanol and water log; BBB,—Blood Brain Barrier; Day 1–21 Q28—once daily for 21 consecutive days followed by 7 days off treatment to comprise a complete cycle of 28 days.

**Table 4:** Dose adjustments suggestions according to concomitant medications and clinical features. Day 1–21 Q28, once daily for 21 consecutive days followed by 7 days off treatment to comprise a complete cycle of 28 days.

Concomitant Medications or Pathophysiological Conditions	Palbociclib 125 mg Once a Day, Day 1–21 Q28	Ribociclib 600 mg Once a Day, Day 1–21 Q28	Abemaciclib 150 mg Twice Daily in Combination with Endocrine Therapy; 200 mg Twice Daily in Monotherapy. Continuous Schedule
Strong CYP3A inhibitor	Avoid. If unavoidable: 75 mg/day starting dose (↓40%) *	Avoid. If unavoidable: 400 mg/day starting dose (↓33.33%) *	Avoid. If unavoidable: 100 mg twice daily starting dose (↓50/33.33%) *
Moderate CYP3A inhibitor	Monitoring	Monitoring	Monitoring
Weak CYP3A inhibitor	Low risk of DDI	Low risk of DDI	Low risk of DDI
Strong CYP3A inducer	Avoid. Consider an alternative	Avoid. Consider an alternative	Avoid. Consider an alternative
Moderate CYP3A inducer	Monitoring	Monitoring	Monitoring
Weak CYP3A inducer	Low risk of DDI	Low risk of DDI	Low risk of DDI
Hepatic impairment recommendation	Child-Pugh A or B: no modifications Child-Pugh C: 75 mg/day starting dose	Child-Pugh A: no modifications Child-Pugh B or C: 400 mg/day starting dose	Child-Pugh A or B: no modifications Child Pugh C: 150 mg/day starting dose
Renal impairment recommendation	Mild to moderate: no modifications Severe or hemodialysis: no data	Mild to moderate: no modifications Severe: lower starting dose to 400 mg/day (EMA) or 200 mg/day starting dose (FDA)	Mild to moderate: no modifications Severe or hemodialysis: no data

DDI—Drug-drug interaction. * The reduction must be maintained during the treatment with the inhibitor and at least five half-lives of elimination after its withdrawal.

## Supplementary Materials

Supplementary materials can be found at https://www.mdpi.com/1422-0067/21/17/6350/s1. Table S1. Co-administered agents categorized according to their potential risk for Drug-Drug interaction (DDI) in combination with CDK4/6 inhibitors (CDKis). Colors suggest the risk of DDI with CDKis: green, low risk DDI; orange, moderate risk DDI; red, high risk DDI.

## References

[1] Bonelli M., La Monica S., Fumarola C., Alfieri R. (2019). Multiple effects of CDK4/6 inhibition in cancer: From cell cycle arrest to immunomodulation. Biochem. Pharmacol..

[2] Spring L.M., Wander S.A., Andre F., Moy B., Turner N.C., Bardia A. (2020). Cyclin-dependent kinase 4 and 6 inhibitors for hormone receptor-positive breast cancer: Past, present, and future. Lancet.

[3] Kotake T., Toi M. (2018). Abemaciclib for the treatment of breast cancer. Expert. Opin. Pharm..

[4] Dickson M.A. (2014). Molecular pathways: CDK4 inhibitors for cancer therapy. Clin. Cancer Res..

[5] Gao J.J., Cheng J., Bloomquist E., Sanchez J., Wedam S.B., Singh H., Amiri-Kordestani L., Ibrahim A., Sridhara R., Goldberg K.B. (2020). CDK4/6 inhibitor treatment for patients with hormone receptor-positive, HER2-negative, advanced or metastatic breast cancer: A US Food and Drug Administration pooled analysis. Lancet Oncol..

[6] Finn R.S., Martin M., Rugo H.S., Jones S., Im S.-A., Gelmon K., Harbeck N., Lipatov O.N., Walshe J.M., Moulder S. (2016). Palbociclib and Letrozole in Advanced Breast Cancer. N. Engl. J. Med..

[7] Hortobagyi G.N., Stemmer S.M., Burris H.A., Yap Y.-S., Sonke G.S., Paluch-Shimon S., Campone M., Blackwell K.L., André F., Winer E.P. (2016). Ribociclib as First-Line Therapy for HR-Positive, Advanced Breast Cancer. N. Engl. J. Med..

[8] Goetz M.P., Toi M., Campone M., Sohn J., Paluch-Shimon S., Huober J., Park I.H., Trédan O., Chen S.-C., Manso L. (2017). MONARCH 3: Abemaciclib As Initial Therapy for Advanced Breast Cancer. J. Clin. Oncol..

[9] Im S.-A., Lu Y.-S., Bardia A., Harbeck N., Colleoni M., Franke F., Chow L., Sohn J., Lee K.-S., Campos-Gomez S. (2019). Overall Survival with Ribociclib plus Endocrine Therapy in Breast Cancer. N. Engl. J. Med..

[10] KISQALI® (ribociclib) (2020). Highlights of Prescribing Information. https://www.accessdata.fda.gov/drugsatfda_docs/label/2019/209092s004lbl.pdf.

[11] Committee for Medicinal Products for Human Use (CHMP) KISQALI® (ribociclib) EPAR Assessment Report Variation. https://www.ema.europa.eu/en/documents/variation-report/kisqali-h-c-4213-ii-0004-epar-assessment-report-variation_en.pdf.

[12] Turner N.C., Slamon D.J., Ro J., Bondarenko I., Im S.-A., Masuda N., Colleoni M., DeMichele A., Loi S., Verma S. (2018). Overall Survival with Palbociclib and Fulvestrant in Advanced Breast Cancer. N. Engl. J. Med..

[13] Slamon D.J., Neven P., Chia S., Fasching P.A., De Laurentiis M., Im S.-A., Petrakova K., Bianchi G.V., Esteva F.J., Martín M. (2020). Overall Survival with Ribociclib plus Fulvestrant in Advanced Breast Cancer. N. Engl. J. Med..

[14] Sledge G.W., Toi M., Neven P., Sohn J., Inoue K., Pivot X., Burdaeva O., Okera M., Masuda N., Kaufman P.A. (2019). The Effect of Abemaciclib Plus Fulvestrant on Overall Survival in Hormone Receptor-Positive, ERBB2-Negative Breast Cancer That Progressed on Endocrine Therapy-MONARCH 2: A Randomized Clinical Trial. JAMA Oncol..

[15] Dickler M.N., Tolaney S.M., Rugo H.S., Cortés J., Diéras V., Patt D., Wildiers H., Hudis C.A., O’Shaughnessy J., Zamora E. (2017). MONARCH 1, A Phase II Study of Abemaciclib, a CDK4 and CDK6 Inhibitor, as a Single Agent, in Patients with Refractory HR+/HER2- Metastatic Breast Cancer. Clin. Cancer Res..

[16] Committee for Medicinal Products for Human Use (CHMP) VERZENIOTM (abemaciclib) EPAR Assessment Report. https://www.ema.europa.eu/en/documents/assessment-report/verzenios-epar-public-assessment-report_en.pdf.

[17] Petrelli F., Ghidini A., Pedersini R., Cabiddu M., Borgonovo K., Parati M.C., Ghilardi M., Amoroso V., Berruti A., Barni S. (2019). Comparative efficacy of palbociclib, ribociclib and abemaciclib for ER+ metastatic breast cancer: An adjusted indirect analysis of randomized controlled trials. Breast Cancer Res. Treat..

[18] Lee K.W.C., Lord S., Finn R.S., Lim E., Martin A., Loi S., Lynch J., Friedlander M., Lee C.K. (2019). The impact of ethnicity on efficacy and toxicity of cyclin D kinase 4/6 inhibitors in advanced breast cancer: A meta-analysis. Breast Cancer Res. Treat..

[19] Pala L., Conforti F., Goldhirsch A. (2020). Ethnicity-based differences in breast cancer features and responsiveness to CDK4/6 inhibitors combined with endocrine therapy. Lancet Oncol..

[20] Fukunaga S., Kusama M., Arnold F.L., Ono S. (2011). Ethnic differences in pharmacokinetics in new drug applications and approved doses in Japan. J. Clin. Pharm..

[21] Arnold F.L., Kusama M., Ono S. (2010). Exploring differences in drug doses between Japan and Western countries. Clin. Pharmacol. Ther..

[22] Warth B., Raffeiner P., Granados A., Huan T., Fang M., Forsberg E.M., Benton H.P., Goetz L., Johnson C.H., Siuzdak G. (2018). Metabolomics Reveals that Dietary Xenoestrogens Alter Cellular Metabolism Induced by Palbociclib/Letrozole Combination Cancer Therapy. Cell Chem. Biol..

[23] Kan Z., Ding Y., Kim J., Jung H.H., Chung W., Lal S., Cho S., Fernandez-Banet J., Lee S.K., Kim S.W. (2018). Multi-omics profiling of younger Asian breast cancers reveals distinctive molecular signatures. Nat. Commun..

[24] Doi T., Hewes B., Kakizume T., Tajima T., Ishikawa N., Yamada Y. (2018). Phase I study of single-agent ribociclib in Japanese patients with advanced solid tumors. Cancer Sci..

[25] Shah R.R., Smith R.L. (2015). Addressing phenoconversion: The Achilles’ heel of personalized medicine. Br. J. Clin. Pharm..

[26] VanArsdale T., Boshoff C., Arndt K.T., Abraham R.T. (2015). Molecular Pathways: Targeting the Cyclin D-CDK4/6 Axis for Cancer Treatment. Clin. Cancer Res..

[27] Law M.E., Corsino P.E., Narayan S., Law B.K. (2015). Cyclin-Dependent Kinase Inhibitors as Anticancer Therapeutics. Mol. Pharmacol..

[28] Shah A., Bloomquist E., Tang S., Fu W., Bi Y., Liu Q., Yu J., Zhao P., Palmby T.R., Goldberg K.B. (2018). FDA Approval: Ribociclib for the Treatment of Postmenopausal Women with Hormone Receptor-Positive, HER2-Negative Advanced or Metastatic Breast Cancer. Clin. Cancer Res..

[29] Sammons S.L., Topping D.L., Blackwell K.L. (2017). HR+, HER2- Advanced Breast Cancer and CDK4/6 Inhibitors: Mode of Action, Clinical Activity, and Safety Profiles. Curr. Cancer Drug Targets.

[30] Poratti M., Marzaro G. (2019). Third-generation CDK inhibitors: A review on the synthesis and binding modes of Palbociclib, Ribociclib and Abemaciclib. Eur. J. Med. Chem..

[31] Hafner M., Mills C.E., Subramanian K., Chen C., Chung M., Boswell S.A., Everley R.A., Liu C., Walmsley C.S., Juric D. (2019). Multi-omics profiling establishes the polypharmacology of FDA Approved CDK4/6 inhibitors and the potential for differential clinical activity. Cell Chem. Biol..

[32] Roberts P.J., Bisi J.E., Strum J.C., Combest A.J., Darr D.B., Usary J.E., Zamboni W.C., Wong K.-K., Perou C.M., Sharpless N.E. (2012). Multiple roles of cyclin-dependent kinase 4/6 inhibitors in cancer therapy. J. Natl. Cancer Inst..

[33] Wood D.J., Endicott J.A. (2018). Structural insights into the functional diversity of the CDK-cyclin family. Open Biol..

[34] Johnson J., Thijssen B., McDermott U., Garnett M., Wessels L.F.A., Bernards R. (2016). Targeting the RB-E2F pathway in breast cancer. Oncogene.

[35] Committee for Medicinal Products for Human Use (CHMP) IBRANCE® (palbociclib) EPAR Assessment Report. https://www.ema.europa.eu/en/documents/assessment-report/piqray-epar-public-assessment-report_en.pdf.

[36] Asghar U., Witkiewicz A.K., Turner N.C., Knudsen E.S. (2015). The history and future of targeting cyclin-dependent kinases in cancer therapy. Nat. Rev. Drug Discov..

[37] Hu M.G., Deshpande A., Schlichting N., Hinds E.A., Mao C., Dose M., Hu G., Van Etten R.A., Gounari F., Hinds P.W. (2011). CDK6 kinase activity is required for thymocyte development. Blood.

[38] Garriga J., Graña X. (2014). CDK9 inhibition strategy defines distinct sets of target genes. BMC Res. Notes.

[39] Tripathy D., Hortobagyi G.N., Chan A., Im S.-A., Chia S., Yardley D., Esteva F.J., Hurvitz S.A., Ridolfi A., Slamon D. (2019). Pooled safety analysis of first-line ribociclib (rib) plus endocrine therapy (ET) in HR+/HER2– advanced breast cancer (ABC). Ann. Oncol..

[40] Johnston S., Martin M., Di Leo A., Im S.-A., Awada A., Forrester T., Frenzel M., Hardebeck M.C., Cox J., Barriga S. (2019). MONARCH 3 final PFS: A randomized study of abemaciclib as initial therapy for advanced breast cancer. NPJ Breast Cancer.

[41] Diéras V., Rugo H.S., Schnell P., Gelmon K., Cristofanilli M., Loi S., Colleoni M., Lu D.R., Mori A., Gauthier E. (2018). Long-term Pooled Safety Analysis of Palbociclib in Combination with Endocrine Therapy for HR+/HER2- Advanced Breast Cancer. J. Natl. Cancer Inst..

[42] Thein K.Z., Htut T.W., Ball S., Swarup S., Sultan A., Oo T.H. (2020). Venous thromboembolism risk in patients with hormone receptor-positive HER2-negative metastatic breast cancer treated with combined CDK 4/6 inhibitors plus endocrine therapy versus endocrine therapy alone: A systematic review and meta-analysis of randomized controlled trials. Breast Cancer Res. Treat..

[43] Undevia S.D., Gomez-Abuin G., Ratain M.J. (2005). Pharmacokinetic variability of anticancer agents. Nat. Rev. Cancer.

[44] Schmidt M. (2016). Palbociclib—From Bench to Bedside and Beyond. Breast Care (Basel).

[45] IBRANCE® (palbociclib) (2020). Highlights of Prescribing Information. https://www.accessdata.fda.gov/drugsatfda_docs/label/2019/207103s008lbl.pdf.

[46] FDA (2020). VERZENIO^TM^ (abemaciclib). Highlights of Prescribing Information. https://www.accessdata.fda.gov/drugsatfda_docs/label/2018/208855s000lbl.pdf.

[47] Chen P., Lee N.V., Hu W., Xu M., Ferre R.A., Lam H., Bergqvist S., Solowiej J., Diehl W., He Y.-A. (2016). Spectrum and Degree of CDK Drug Interactions Predicts Clinical Performance. Mol. Cancer Ther..

[48] Flaherty K.T., LoRusso P.M., DeMichele A., Abramson V.G., Courtney R., Randolph S.S., Shaik M.N., Wilner K.D., O’Dwyer P.J., Schwartz G.K. (2012). Phase I, Dose-Escalation Trial of the Oral Cyclin-Dependent Kinase 4/6 Inhibitor PD 0332991, Administered Using a 21-Day Schedule in Patients with Advanced Cancer. Clin. Cancer Res..

[49] Infante J.R., Cassier P.A., Gerecitano J.F., Witteveen P.O., Chugh R., Ribrag V., Chakraborty A., Matano A., Dobson J.R., Crystal A.S. (2016). A Phase I Study of the Cyclin-Dependent Kinase 4/6 Inhibitor Ribociclib (LEE011) in Patients with Advanced Solid Tumors and Lymphomas. Clin. Cancer Res..

[50] Bellet M., Ahmad F., Villanueva R., Valdivia C., Palomino-Doza J., Ruiz A., Gonzàlez X., Adrover E., Azaro A., Valls-Margarit M. (2019). Palbociclib and ribociclib in breast cancer: Consensus workshop on the management of concomitant medication. Adv. Med. Oncol.

[51] Raub T.J., Wishart G.N., Kulanthaivel P., Staton B.A., Ajamie R.T., Sawada G.A., Gelbert L.M., Shannon H.E., Sanchez-Martinez C., De Dios A. (2015). Brain Exposure of Two Selective Dual CDK4 and CDK6 Inhibitors and the Antitumor Activity of CDK4 and CDK6 Inhibition in Combination with Temozolomide in an Intracranial Glioblastoma Xenograft. Drug Metab. Dispos..

[52] Patel Y.T., Davis A., Baker S.J., Campagne O., Stewart C.F. (2019). CNS penetration of the CDK4/6 inhibitor ribociclib in non-tumor bearing mice and mice bearing pediatric brain tumors. Cancer Chemother. Pharmacol..

[53] Martínez-Chávez A., van Hoppe S., Rosing H., Lebre M.C., Tibben M., Beijnen J.H., Schinkel A.H. (2019). P-glycoprotein Limits Ribociclib Brain Exposure and CYP3A4 Restricts Its Oral Bioavailability. Mol. Pharm..

[54] Chong Q.-Y., Kok Z.-H., Bui N.-L.-C., Xiang X., Wong A.L.-A., Yong W.-P., Sethi G., Lobie P.E., Wang L., Goh B.-C. (2020). A unique CDK4/6 inhibitor: Current and future therapeutic strategies of abemaciclib. Pharmacol. Res..

[55] Rascon K., Flajc G., De Angelis C., Liu X., Trivedi M.V., Ekinci E. (2019). Ribociclib in HR+/HER2- Advanced or Metastatic Breast Cancer Patients. Ann. Pharm..

[56] Ruiz-Garcia A., Plotka A., O’Gorman M., Wang D.D. (2017). Effect of food on the bioavailability of palbociclib. Cancer Chemother. Pharmacol..

[57] Tate S.C., Sykes A.K., Kulanthaivel P., Chan E.M., Turner P.K., Cronier D.M. (2018). A Population Pharmacokinetic and Pharmacodynamic Analysis of Abemaciclib in a Phase I Clinical Trial in Cancer Patients. Clin. Pharm..

[58] Curigliano G., Criscitiello C., Esposito A., Intra M., Minucci S. (2017). Pharmacokinetic drug evaluation of ribociclib for the treatment of metastatic, hormone-positive breast cancer. Expert Opin. Drug Metab. Toxicol..

[59] Sledge G.W., Toi M., Neven P., Sohn J., Inoue K., Pivot X., Burdaeva O., Okera M., Masuda N., Kaufman P.A. (2017). MONARCH 2: Abemaciclib in Combination With Fulvestrant in Women With HR+/HER2- Advanced Breast Cancer Who Had Progressed While Receiving Endocrine Therapy. J. Clin. Oncol..

[60] Sonke G.S., Hart L.L., Campone M., Erdkamp F., Janni W., Verma S., Villanueva C., Jakobsen E., Alba E., Wist E. (2018). Ribociclib with letrozole vs. letrozole alone in elderly patients with hormone receptor-positive, HER2-negative breast cancer in the randomized MONALEESA-2 trial. Breast Cancer Res. Treat..

[61] Qin X., Wang X. (2019). Role of vitamin D receptor in the regulation of CYP3A gene expression. Acta Pharm. Sin. B.

[62] James M.O., Ambadapadi S. (2013). Interactions of cytosolic sulfotransferases with xenobiotics. Drug Metab. Rev..

[63] Marto N., Morello J., Monteiro E.C., Pereira S.A. (2017). Implications of sulfotransferase activity in interindividual variability in drug response: Clinical perspective on current knowledge. Drug Metab. Rev..

[64] Fukushima-Uesaka H., Saito Y., Watanabe H., Shiseki K., Saeki M., Nakamura T., Kurose K., Sai K., Komamura K., Ueno K. (2004). Haplotypes of CYP3A4 and their close linkage with CYP3A5 haplotypes in a Japanese population. Hum. Mutat..

[65] Lamba J.K., Lin Y.S., Schuetz E.G., Thummel K.E. (2002). Genetic contribution to variable human CYP3A-mediated metabolism. Adv. Drug Deliv. Rev..

[66] Park J.-E., Kim K.-B., Bae S.K., Moon B.-S., Liu K.-H., Shin J.-G. (2008). Contribution of cytochrome P450 3A4 and 3A5 to the metabolism of atorvastatin. Xenobiotica.

[67] Werk A.N., Cascorbi I. (2014). Functional gene variants of CYP3A4. Clin. Pharmacol. Ther..

[68] Wang D., Guo Y., Wrighton S.A., Cooke G.E., Sadee W. (2011). Intronic polymorphism in CYP3A4 affects hepatic expression and response to statin drugs. Pharm. J..

[69] Baxter S.D., Teft W.A., Choi Y.-H., Winquist E., Kim R.B. (2014). Tamoxifen-associated hot flash severity is inversely correlated with endoxifen concentration and CYP3A4*22. Breast Cancer Res. Treat..

[70] Teft W.A., Gong I.Y., Dingle B., Potvin K., Younus J., Vandenberg T.A., Brackstone M., Perera F.E., Choi Y.-H., Zou G. (2013). CYP3A4 and seasonal variation in vitamin D status in addition to CYP2D6 contribute to therapeutic endoxifen level during tamoxifen therapy. Breast Cancer Res. Treat..

[71] Diekstra M.H.M., Klümpen H.J., Lolkema M.P.J.K., Yu H., Kloth J.S.L., Gelderblom H., van Schaik R.H.N., Gurney H., Swen J.J., Huitema A.D.R. (2014). Association analysis of genetic polymorphisms in genes related to sunitinib pharmacokinetics, specifically clearance of sunitinib and SU12662. Clin. Pharmacol. Ther..

[72] Kreutz R.P., Owens J., Jin Y., Nystrom P., Desta Z., Kreutz Y., Breall J.A., Li L., Chiang C., Kovacs R.J. (2013). Cytochrome P450 3A4*22, PPAR-α, and ARNT polymorphisms and clopidogrel response. Clin. Pharm..

[73] Shi Y., Li Y., Tang J., Zhang J., Zou Y., Cai B., Wang L. (2013). Influence of CYP3A4, CYP3A5 and MDR-1 polymorphisms on tacrolimus pharmacokinetics and early renal dysfunction in liver transplant recipients. Gene.

[74] Miao J., Jin Y., Marunde R.L., Gorski C.J., Kim S., Quinney S., Radovich M., Li L., Hall S.D. (2009). Association of genotypes of the CYP3A cluster with midazolam disposition In Vivo. Pharm. J..

[75] Tremmel R., Klein K., Battke F., Fehr S., Winter S., Scheurenbrand T., Schaeffeler E., Biskup S., Schwab M., Zanger U.M. (2020). Copy number variation profiling in pharmacogenes using panel-based exome resequencing and correlation to human liver expression. Hum. Genet..

[76] Gordon A.S., Tabor H.K., Johnson A.D., Snively B.M., Assimes T.L., Auer P.L., Ioannidis J.P.A., Peters U., Robinson J.G., Sucheston L.E. (2014). Quantifying rare, deleterious variation in 12 human cytochrome P450 drug-metabolism genes in a large-scale exome dataset. Hum. Mol. Genet..

[77] Ingelman-Sundberg M., Mkrtchian S., Zhou Y., Lauschke V.M. (2018). Integrating rare genetic variants into pharmacogenetic drug response predictions. Hum. Genom..

[78] Westlind-Johnsson A., Hermann R., Huennemeyer A., Hauns B., Lahu G., Nassr N., Zech K., Ingelman-Sundberg M., von Richter O. (2006). Identification and characterization of CYP3A4*20, a novel rare CYP3A4 allele without functional activity. Clin. Pharmacol. Ther..

[79] Werk A.N., Lefeldt S., Bruckmueller H., Hemmrich-Stanisak G., Franke A., Roos M., Küchle C., Steubl D., Schmaderer C., Bräsen J.H. (2014). Identification and characterization of a defective CYP3A4 genotype in a kidney transplant patient with severely diminished tacrolimus clearance. Clin. Pharmacol. Ther..

[80] Birdwell K.A., Decker B., Barbarino J.M., Peterson J.F., Stein C.M., Sadee W., Wang D., Vinks A.A., He Y., Swen J.J. (2015). Clinical Pharmacogenetics Implementation Consortium (CPIC) Guidelines for CYP3A5 Genotype and Tacrolimus Dosing. Clin. Pharmacol. Ther..

[81] García-Anguita A., Ortega L., Garcés C. (2013). Relationship between polymorphisms in the sulfotransferase SULT2A1 gene and dehydroepiandrosterone sulfate concentration in children. Exp. Biol. Med. (Maywood).

[82] Schulze J., Johansson M., Thörngren J.-O., Garle M., Rane A., Ekström L. (2013). SULT2A1 Gene Copy Number Variation is Associated with Urinary Excretion Rate of Steroid Sulfates. Front. Endocrinol. (Lausanne).

[83] Drocourt L., Ourlin J.-C., Pascussi J.-M., Maurel P., Vilarem M.-J. (2002). Expression of CYP3A4, CYP2B6, and CYP2C9 is regulated by the vitamin D receptor pathway in primary human hepatocytes. J. Biol. Chem..

[84] De Mattia E., Cecchin E., Roncato R., Toffoli G. (2016). Pregnane X receptor, constitutive androstane receptor and hepatocyte nuclear factors as emerging players in cancer precision medicine. Pharmacogenomics.

[85] Elens L., Capron A., van Schaik R.H.N., De Meyer M., De Pauw L., Eddour D.C., Latinne D., Wallemacq P., Mourad M., Haufroid V. (2013). Impact of CYP3A4*22 allele on tacrolimus pharmacokinetics in early period after renal transplantation: Toward updated genotype-based dosage guidelines. Drug Monit..

[86] Leschziner G., Zabaneh D., Pirmohamed M., Owen A., Rogers J., Coffey A.J., Balding D.J., Bentley D.B., Johnson M.R. (2006). Exon sequencing and high resolution haplotype analysis of ABC transporter genes implicated in drug resistance. Pharmacogenet. Genom..

[87] Salama N.N., Yang Z., Bui T., Ho R.J.Y. (2006). MDR1 haplotypes significantly minimize intracellular uptake and transcellular P-gp substrate transport in recombinant LLC-PK1 cells. J. Pharm. Sci..

[88] Takahashi N., Wakita H., Miura M., Scott S.A., Nishii K., Masuko M., Sakai M., Maeda Y., Ishige K., Kashimura M. (2010). Correlation between imatinib pharmacokinetics and clinical response in Japanese patients with chronic-phase chronic myeloid leukemia. Clin. Pharmacol. Ther..

[89] Li J., Cusatis G., Brahmer J., Sparreboom A., Robey R.W., Bates S.E., Hidalgo M., Baker S.D. (2007). Association of variant ABCG2 and the pharmacokinetics of epidermal growth factor receptor tyrosine kinase inhibitors in cancer patients. Cancer Biol. Ther..

[90] Mizuno T., Terada T., Kamba T., Fukudo M., Katsura T., Nakamura E., Ogawa O., Inui K. (2010). ABCG2 421C>A polymorphism and high exposure of sunitinib in a patient with renal cell carcinoma. Ann. Oncol..

[91] Fujita K., Hirose T., Kusumoto S., Sugiyama T., Shirai T., Nakashima M., Akiyama Y., Sasaki Y. (2014). High exposure to erlotinib and severe drug-induced interstitial lung disease in patients with non-small-cell lung cancer. Lung Cancer.

[92] Rudin C.M., Liu W., Desai A., Karrison T., Jiang X., Janisch L., Das S., Ramirez J., Poonkuzhali B., Schuetz E. (2008). Pharmacogenomic and pharmacokinetic determinants of erlotinib toxicity. J. Clin. Oncol..

[93] Reyner E., Lum B., Jing J., Kagedal M., Ware J.A., Dickmann L.J. (2020). Intrinsic and Extrinsic Pharmacokinetic Variability of Small Molecule Targeted Cancer Therapy. Clin. Transl. Sci..

[94] Kelly C.M., Juurlink D.N., Gomes T., Duong-Hua M., Pritchard K.I., Austin P.C., Paszat L.F. (2010). Selective serotonin reuptake inhibitors and breast cancer mortality in women receiving tamoxifen: A population based cohort study. BMJ.

[95] Schaffner F., Poper H. (1963). Capillarization of hepatic sinusoids in man. Gastroenterology.

[96] Van Beers B.E., Materne R., Annet L., Hermoye L., Sempoux C., Peeters F., Smith A.M., Jamart J., Horsmans Y. (2003). Capillarization of the sinusoids in liver fibrosis: Noninvasive assessment with contrast-enhanced MRI in the rabbit. Magn. Reason. Med..

[97] Vuppalanchi R., Saxena R., Storniolo A.M.V., Chalasani N. (2017). Pseudocirrhosis and liver failure in patients with metastatic breast cancer after treatment with palbociclib. Hepatology.

[98] Schlotman A., Stater A., Schuler K., Heideman J., Abramson V. (2020). Grade 3 Hepatotoxicity following Fulvestrant, Palbociclib, and Erdafitinib Therapy in a Patient with ER-Positive/PR-Negative/HER2-Negative Metastatic Breast Cancer: A Case Report. Case Rep. Oncol..

[99] Atallah R., Parker N.A., Hamouche K., Truong Q.V., Dingwall M. (2020). Palbociclib-Induced Liver Failure. Kans J. Med..

[100] EMA Kisqali Procedural Steps Taken and Scientific Information after the Authorisation 2020. https://www.ema.europa.eu/en/documents/procedural-steps-after/kisqali-epar-procedural-steps-taken-scientific-information-after-authorisation_en.pdf.

[101] Kim E.S. (2017). Abemaciclib: First Global Approval. Drugs.

[102] Freire A.C., Basit A.W., Choudhary R., Piong C.W., Merchant H.A. (2011). Does sex matter? The influence of gender on gastrointestinal physiology and drug delivery. Int. J. Pharm..

[103] Samant T.S., Dhuria S., Lu Y., Laisney M., Yang S., Grandeury A., Mueller-Zsigmondy M., Umehara K., Huth F., Miller M. (2018). Ribociclib Bioavailability Is Not Affected by Gastric pH Changes or Food Intake: In Silico and Clinical Evaluations. Clin. Pharmacol. Ther..

[104] Paine M.F., Ludington S.S., Chen M.-L., Stewart P.W., Huang S.-M., Watkins P.B. (2005). Do men and women differ in proximal small intestinal CYP3A or P-glycoprotein expression?. Drug Metab. Dispos..

[105] MacLean C., Moenning U., Reichel A., Fricker G. (2008). Closing the gaps: A full scan of the intestinal expression of p-glycoprotein, breast cancer resistance protein, and multidrug resistance-associated protein 2 in male and female rats. Drug Metab. Dispos..

[106] Wolbold R., Klein K., Burk O., Nüssler A.K., Neuhaus P., Eichelbaum M., Schwab M., Zanger U.M. (2003). Sex is a major determinant of CYP3A4 expression in human liver. Hepatology.

[107] Merino G., van Herwaarden A.E., Wagenaar E., Jonker J.W., Schinkel A.H. (2005). Sex-dependent expression and activity of the ATP-binding cassette transporter breast cancer resistance protein (BCRP/ABCG2) in liver. Mol. Pharmacol..

[108] Makkar R.R., Fromm B.S., Steinman R.T., Meissner M.D., Lehmann M.H. (1993). Female gender as a risk factor for torsades de pointes associated with cardiovascular drugs. JAMA.

[109] Rodriguez I., Kilborn M.J., Liu X.K., Pezzullo J.C., Woosley R.L. (2001). Drug-induced QT prolongation in women during the menstrual cycle. JAMA.

[110] Mitchell S.C., Smith R.L., Waring R.H. (2009). The menstrual cycle and drug metabolism. Curr. Drug Metab..

[111] Ximenez J.P.B., de Andrade J.M., Marques M.P., Coelho E.B., Suarez-Kurtz G., Lanchote V.L. (2019). Hormonal status affects plasma exposure of tamoxifen and its main metabolites in tamoxifen-treated breast cancer patients. BMC Pharm. Toxicol..

[112] Kanakis G.A., Jørgensen N., Goulis D.G. (2018). Breast Cancer in Men. N. Engl. J. Med..

[113] Frisone D., Charrier M., Clement S., Christinat Y., Thouvenin L., Homicsko K., Michielin O., Bodmer A., Chappuis P.O., McKee T.A. (2019). Durable response to palbociclib and letrozole in ovarian cancer with CDKN2A loss. Cancer Biol..

[114] Morgan E.T., Goralski K.B., Piquette-Miller M., Renton K.W., Robertson G.R., Chaluvadi M.R., Charles K.A., Clarke S.J., Kacevska M., Liddle C. (2008). Regulation of drug-metabolizing enzymes and transporters in infection, inflammation, and cancer. Drug Metab. Dispos..

[115] Slaviero K.A., Clarke S.J., Rivory L.P. (2003). Inflammatory response: An unrecognised source of variability in the pharmacokinetics and pharmacodynamics of cancer chemotherapy. Lancet Oncol..

[116] Garattini S. (2007). Pharmacokinetics in cancer chemotherapy. Eur. J. Cancer.

[117] Abdel-Razzak Z., Loyer P., Fautrel A., Gautier J.C., Corcos L., Turlin B., Beaune P., Guillouzo A. (1993). Cytokines down-regulate expression of major cytochrome P-450 enzymes in adult human hepatocytes in primary culture. Mol. Pharmacol..

[118] Aitken A.E., Morgan E.T. (2007). Gene-specific effects of inflammatory cytokines on cytochrome P450 2C, 2B6 and 3A4 mRNA levels in human hepatocytes. Drug Metab. Dispos..

[119] Sunman J.A., Hawke R.L., LeCluyse E.L., Kashuba A.D.M. (2004). Kupffer cell-mediated IL-2 suppression of CYP3A activity in human hepatocytes. Drug Metab. Dispos..

[120] Moriya N., Kataoka H., Fujino H., Nishikawa J., Kugawa F. (2014). Different expression patterns of hepatic cytochrome P450 s during anaphylactic or lipopolysaccharide-induced inflammation. Pharmazie.

[121] Morgan E.T., Dempsey J.L., Mimche S.M., Lamb T.J., Kulkarni S., Cui J.Y., Jeong H., Slitt A.L. (2018). Physiological Regulation of Drug Metabolism and Transport: Pregnancy, Microbiome, Inflammation, Infection, and Fasting. Drug Metab. Dispos..

[122] Rivory L.P., Slaviero K.A., Clarke S.J. (2002). Hepatic cytochrome P450 3A drug metabolism is reduced in cancer patients who have an acute-phase response. Br. J. Cancer.

[123] Noll E.M., Eisen C., Stenzinger A., Espinet E., Muckenhuber A., Klein C., Vogel V., Klaus B., Nadler W., Rösli C. (2016). CYP3A5 mediates basal and acquired therapy resistance in different subtypes of pancreatic ductal adenocarcinoma. Nat. Med..

[124] Schmidt R., Baumann F., Knüpfer H., Brauckhoff M., Horn L.-C., Schönfelder M., Köhler U., Preiss R. (2004). CYP3A4, CYP2C9 and CYP2B6 expression and ifosfamide turnover in breast cancer tissue microsomes. Br. J. Cancer.

[125] Waghray D., Zhang Q. (2018). Inhibit or Evade Multidrug Resistance P-Glycoprotein in Cancer Treatment. J. Med. Chem..

[126] Kim M.S., Shigenaga J., Moser A., Grunfeld C., Feingold K.R. (2004). Suppression of DHEA sulfotransferase (Sult2A1) during the acute-phase response. Am. J. Physiol. Endocrinol. Metab..

[127] Lin N.U., Amiri-Kordestani L., Palmieri D., Liewehr D.J., Steeg P.S. (2013). CNS metastases in breast cancer: Old challenge, new frontiers. Clin. Cancer Res..

[128] Larochelle C., Alvarez J.I., Prat A. (2011). How do immune cells overcome the blood-brain barrier in multiple sclerosis?. FEBS Lett..

[129] Henson J.W., Cordon-Cardo C., Posner J.B. (1992). P-glycoprotein expression in brain tumors. J. Neuro-Oncol..

[130] Patnaik A., Rosen L.S., Tolaney S.M., Tolcher A.W., Goldman J.W., Gandhi L., Papadopoulos K.P., Beeram M., Rasco D.W., Hilton J.F. (2016). Efficacy and Safety of Abemaciclib, an Inhibitor of CDK4 and CDK6, for Patients with Breast Cancer, Non-Small Cell Lung Cancer, and Other Solid Tumors. Cancer Discov..

[131] Anders C.K., Le Rhun E., Bachelot T.D., Yardley D.A., Awada A., Conte P.F., Kabos P., Bear M., Yang Z., Chen Y. (2019). A phase II study of abemaciclib in patients (pts) with brain metastases (BM) secondary to HR+, HER2− metastatic breast cancer (MBC). J. Clin. Oncol..

[132] Tien A.-C., Li J., Bao X., Derogatis A., Kim S., Mehta S., Sanai N. (2019). A Phase 0 Trial of Ribociclib in Recurrent Glioblastoma Patients Incorporating a Tumor Pharmacodynamic- and Pharmacokinetic-Guided Expansion Cohort. Clin. Cancer Res..

[133] Brastianos P., Cohen J., Wang N., Lee E., Ligibel J., Chukwueke U., Keeley M., Oh K., Whie M., Gerstner E. A phase II study of palbociclib in progressive brain metastases harboring alterations in the CDK pathway. Proceedings of the 4th Annual Meeting and Education Day.

[134] Bloomgarden Z.T. (2005). Second World Congress on the Insulin Resistance Syndrome: Insulin resistance syndrome and nonalcoholic fatty liver disease. Diabetes Care.

[135] Protani M., Coory M., Martin J.H. (2010). Effect of obesity on survival of women with breast cancer: Systematic review and meta-analysis. Breast Cancer Res. Treat..

[136] Amadou A., Ferrari P., Muwonge R., Moskal A., Biessy C., Romieu I., Hainaut P. (2013). Overweight, obesity and risk of premenopausal breast cancer according to ethnicity: A systematic review and dose-response meta-analysis. Obes. Rev..

[137] Begum P., Richardson C.E., Carmichael A.R. (2009). Obesity in post menopausal women with a family history of breast cancer: Prevalence and risk awareness. Int. Semin. Surg. Oncol..

[138] Ayoub N.M., Yaghan R.J., Abdo N.M., Matalka I.I., Akhu-Zaheya L.M., Al-Mohtaseb A.H. (2019). Impact of Obesity on Clinicopathologic Characteristics and Disease Prognosis in Pre- and Postmenopausal Breast Cancer Patients: A Retrospective Institutional Study. J. Obes..

[139] Committee for Human Medicinal Products (CHMP) Reflection Paper on Investigation of Pharmacokinetics and Pharmacodynamics in the Obese Population. https://www.ema.europa.eu/en/documents/scientific-guideline/reflection-paper-investigation-pharmacokinetics-pharmacodynamics-obese-population_en.pdf.

[140] Evans M.A., Triggs E.J., Cheung M., Broe G.A., Creasey H. (1981). Gastric emptying rate in the elderly: Implications for drug therapy. J. Am. Geriatr Soc..

[141] Rubinow D.R., Moore M. (2004). Sex-dependent modulation of treatment response. Dialogues Clin. Neurosci..

[142] Robertson D.R., Waller D.G., Renwick A.G., George C.F. (1988). Age-related changes in the pharmacokinetics and pharmacodynamics of nifedipine. Br. J. Clin. Pharm..

[143] Greenblatt D.J., Harmatz J.S., Shapiro L., Engelhardt N., Gouthro T.A., Shader R.I. (1991). Sensitivity to triazolam in the elderly. N. Engl. J. Med..

[144] Evans C. (2005). Malnutrition in the elderly: A multifactorial failure to thrive. Perm. J..

[145] van Assema D.M.E., Lubberink M., Bauer M., van der Flier W.M., Schuit R.C., Windhorst A.D., Comans E.F.I., Hoetjes N.J., Tolboom N., Langer O. (2012). Blood-brain barrier P-glycoprotein function in Alzheimer’s disease. Brain.

[146] Tesarova P. (2012). Breast cancer in the elderly-Should it be treated differently?. Rep. Pr. Oncol. Radiother..

[147] Portman N., Alexandrou S., Carson E., Wang S., Lim E., Caldon C.E. (2019). Overcoming CDK4/6 inhibitor resistance in ER-positive breast cancer. Endocr. Relat. Cancer.

[148] Pandey K., An H.-J., Kim S.K., Lee S.A., Kim S., Lim S.M., Kim G.M., Sohn J., Moon Y.W. (2019). Molecular mechanisms of resistance to CDK4/6 inhibitors in breast cancer: A review. Int. J. Cancer.

[149] The I., Ruijtenberg S., Bouchet B.P., Cristobal A., Prinsen M.B.W., van Mourik T., Koreth J., Xu H., Heck A.J.R., Akhmanova A. (2015). Rb and FZR1/Cdh1 determine CDK4/6-cyclin D requirement in C. elegans and human cancer cells. Nat. Commun..

[150] Schachter M.M., Merrick K.A., Larochelle S., Hirschi A., Zhang C., Shokat K.M., Rubin S.M., Fisher R.P. (2013). A Cdk7-Cdk4 T-loop phosphorylation cascade promotes G1 progression. Mol. Cell.

[151] Zupkovitz G., Grausenburger R., Brunmeir R., Senese S., Tischler J., Jurkin J., Rembold M., Meunier D., Egger G., Lagger S. (2010). The cyclin-dependent kinase inhibitor p21 is a crucial target for histone deacetylase 1 as a regulator of cellular proliferation. Mol. Cell. Biol..

[152] Efeyan A., Ortega-Molina A., Velasco-Miguel S., Herranz D., Vassilev L.T., Serrano M. (2007). Induction of p53-dependent senescence by the MDM2 antagonist nutlin-3a in mouse cells of fibroblast origin. Cancer Res..

[153] McCartney A., Migliaccio I., Bonechi M., Biagioni C., Romagnoli D., De Luca F., Galardi F., Risi E., De Santo I., Benelli M. (2019). Mechanisms of Resistance to CDK4/6 Inhibitors: Potential Implications and Biomarkers for Clinical Practice. Front. Oncol..

[154] Chen X., Xu D., Li X., Zhang J., Xu W., Hou J., Zhang W., Tang J. (2019). Latest Overview of the Cyclin-Dependent Kinases 4/6 Inhibitors in Breast Cancer: The Past, the Present and the Future. J. Cancer.

[155] Marra A., Curigliano G. (2019). Are all cyclin-dependent kinases 4/6 inhibitors created equal?. NPJ Breast Cancer.

[156] Gervaso L., Montero A.J., Jia X., Khorana A.A. (2020). Venous thromboembolism in breast cancer patients receiving cyclin-dependent kinase inhibitors. J. Thromb. Haemost..

[157] Finn R.S., Crown J.P., Lang I., Boer K., Bondarenko I.M., Kulyk S.O., Ettl J., Patel R., Pinter T., Schmidt M. (2015). The cyclin-dependent kinase 4/6 inhibitor palbociclib in combination with letrozole versus letrozole alone as first-line treatment of oestrogen receptor-positive, HER2-negative, advanced breast cancer (PALOMA-1/TRIO-18): A randomised phase 2 study. Lancet Oncol..

[158] Cheng C.K., Gustafson W.C., Charron E., Houseman B.T., Zunder E., Goga A., Gray N.S., Pollok B., Oakes S.A., James C.D. (2012). Dual blockade of lipid and cyclin-dependent kinases induces synthetic lethality in malignant glioma. Proc. Natl. Acad. Sci. USA.

[159] Matutino A., Amaro C., Verma S. (2018). CDK4/6 inhibitors in breast cancer: Beyond hormone receptor-positive HER2-negative disease. Adv. Med. Oncol..

[160] DeMichele A., Clark A.S., Tan K.S., Heitjan D.F., Gramlich K., Gallagher M., Lal P., Feldman M., Zhang P., Colameco C. (2015). CDK 4/6 inhibitor palbociclib (PD0332991) in Rb+ advanced breast cancer: Phase II activity, safety, and predictive biomarker assessment. Clin. Cancer Res..

[161] Schettini F., De Santo I., Rea C.G., De Placido P., Formisano L., Giuliano M., Arpino G., De Laurentiis M., Puglisi F., De Placido S. (2018). CDK 4/6 Inhibitors as Single Agent in Advanced Solid Tumors. Front. Oncol..

[162] Chou A., Froio D., Nagrial A.M., Parkin A., Murphy K.J., Chin V.T., Wohl D., Steinmann A., Stark R., Drury A. (2018). Tailored first-line and second-line CDK4-targeting treatment combinations in mouse models of pancreatic cancer. Gut.

[163] Teh J.L.F., Aplin A.E. (2019). Arrested Developments: CDK4/6 Inhibitor Resistance and Alterations in the Tumor Immune Microenvironment. Clin. Cancer Res..

[164] Sánchez-Martínez C., Lallena M.J., Sanfeliciano S.G., de Dios A. (2019). Cyclin dependent kinase (CDK) inhibitors as anticancer drugs: Recent advances (2015-2019). Bioorg. Med. Chem. Lett..

[165] Es K., Ak W. (2017). The Strange Case of CDK4/6 Inhibitors: Mechanisms, Resistance, and Combination Strategies. Trends Cancer.

[166] O’Shaughnessy J., Thaddeus Beck J., Royce M. (2018). Everolimus-based combination therapies for HR+, HER2- metastatic breast cancer. Cancer Treat. Rev..

