# Supplementary material for: CDK4/6 Inhibitors in Breast Cancer Treatment: Potential Interactions with Drug, Gene, and Pathophysiological Conditions
Source: Int J Mol Sci. 2020 Sep 1;21(17):6350. doi: 10.3390/ijms21176350 (PMC7504705; doi:10.3390/ijms21176350)
Supplement: Supplementary file 1 [file ijms-21-06350-s001.pdf]

International Journal of *Molecular Sciences*

MDPI

Review

CDK4/6 Inhibitors in Breast Cancer Treatment: Potential Interactions with Drug, Gene and Pathophysiological Conditions

Rossana Roncato [1,*,†], Jacopo Angelini [2,†], Arianna Pani [2,3,†], Erika Cecchin [1], Andrea Sartore-Bianchi [2,4], Salvatore Siena [2,4], Elena De Mattia [1], Francesco Scaglione [2,3,‡] and Giuseppe Toffoli [1,‡]

1 [1] Experimental and Clinical Pharmacology Unit, Centro di Riferimento Oncologico (CRO), IRCCS, 33081 Aviano, Italy; ececchin@cro.it (E.C.); edemattia@cro.it (E.D.M.); gtoffoli@cro.it (G.T.)

2 Department of Oncology and Hemato-Oncology, Università degli Studi di Milano, 20122 Milan, Italy; jacopoangelini1@gmail.com (J.A.); arianna.pani@unimi.it (A.P.); andrea.sartorebianchi@ospedaleniguarda.it (A.S-B.); salvatore.siena@ospedaleniguarda.it (S.S.); francesco.scaglione@unimi.it (F.S.)

3 Clinical Pharmacology Unit, ASST Grande Ospedale Metropolitano Niguarda, Piazza dell'Ospedale Maggiore 3, 20162 Milan, Italy

4 Department of Hematology and Oncology, Niguarda Cancer Center, Grande Ospedale Metropolitano Niguarda, 20162 Milan, Italy

* Correspondence: rroncato@cro.it; Tel.:+390434659130

† These authors contributed equally.

‡ These authors share senior authorship.

Table S1. Co-administered agents categorized according to their potential risk for Drug-Drug interaction (DDI) in combination with CDK4/6 inhibitors (CDKis). Colors suggest the risk of DDI with CDKis: green, low risk DDI; orange, moderate risk DDI; red, high risk DDI. ADME, absorption, distribution, metabolism, and excretion; GI, Gastrointestinal; TdP, Torsades de Pointes; NTI, narrow therapeutic index. * Cardiological toxicity should be considered especially for ribociclib due to the QT prolongation. Modified table from Bellet et al. 2019 [50].

Co-administered agent (Class)		Co-administered agents (Name)	ADME DDI with Palbociclib, Ribociclib and Abemaciclib	Non ADME DDI (TdP) (Caution Should Be Exercised in Combination with Ribociclib *)	Effect of CDKis on Co-Administered Agents
Anti-infective	Antibiotics	Beta-lactams; Tetracyclines; Fosfomycin; Linezolid; Clindamycin; Glycopeptides; Aminoglucosides, Daptomycin.	Low risk DDI	Low risk DDI	-
		Trimetoprim/sulfamethoxazole; Macrolides (azithromycin); Fluoroquinolones (levofloxacin, moxifloxacin, norfloxacin, ofloxacin); Metronidazole	Trimethoprim major CYP3A4 substrate; Azithromycin, levofloxacin and moxifloxacin known TdP risk; Norfloxacin and ofloxacin possible TdP risk; Metronidazole conditional TdP risk	Trimethoprim major CYP3A4 substrate; Azithromycin, levofloxacin and moxifloxacin known TdP risk; Norfloxacin and ofloxacin possible TdP risk; Metronidazole conditional TdP risk	Ribociclib can increase concentration of trimethoprim, with bone marrow toxicity
		Macrolides (erythromycin, claritrhromycin); Rifampicin; Fluoroquinolones (ciprofloxacin)	Moderate to strong CYP3A4 inhibitors/inducers (quasi-irreversible inhibition for macrolides)	Moderate to strong CYP3A4 inhibitors/inducers (quasi-irreversible inhibition for macrolides)	High risk DDI (major CYP3A4 substrate)
	Antiherpetic	Acyclovir, famciclovir, valacyclovir, brivudine, ganciclovir, valganciclovir	Low risk DDI	Low risk DDI	-
	Flu	Oseltamivir	Low risk DDI	Low risk DDI	-
	HIV, hepatitis	Nucleoside analog reverse transcriptase inhibitors (lamivudine, abacavir, tenofivir) Integrase inhibitors (dolutegravir, raltegravir)	Low risk DDI	Low risk DDI	-
		Non-nucleoside reverse transcriptase inhibitors: nevirapine	Major substrate and weak CYP3A4 inducer	Major substrate and weak CYP3A4 inducer	Major CYP3A4 substrate
		Protease inhibitors for HIV and HCV (atazanavir, darunavir, lopinavir, indinavir, ritonavir (usually administered in combination with	Moderate to strong CYP3A4 inhibitors/inducers	Moderate to strong CYP3A4 inhibitors/inducers	High risk DDI (Major CYP3A4 substrate)

		ritonavir); Non-nucleoside reverse transcriptase inhibitors: efavirenz			
	Antifungal	Amphotericin B	Low risk DDI	Amphotericin B with conditional TdP risk, caution should be exercised in combination with ribociclib.	Low risk DDI
		Echinocandins (caspofungin, anidulafungin, micafungin)	Low risk DDI	Low risk DDI	-
		Fluconazole, itraconazole, isavuconazole, posaconazole, voriconazole, ketoconazole	Moderate to strong CYP3A4 inhibitors	Moderate to strong CYP3A4 inhibitors	High risk DDI (Major CYP3A4 substrate)
GI treatment	Antiemetics	Metoclopramide, olanzapine, palonosetron, granisetron	Low risk DDI	Conditional to possible TdP risk	Low risk DDI
		Rolapitant, fosaprepitant, dexamethasone	Weak CYP3A4 inducer/inhibitor of CYP3A4	Weak CYP3A4 inducer/inhibitor of CYP3A4	Major substrates
		Aprepitant, netupitant, ondansetron, domperidone	Moderate CYP3A4 inhibitor.	Moderate CYP3A4 inhibitor	Major substrates
	Antacids and gastric mucosal protective	Ranitidine, famotidine, alluminium hydroxide, bismuth subsalicylate, zinc acexamate, sucralfate	Low risk DDI	Low risk DDI	-
		Esomeprazole, omeprazole, pantoprazole	Low risk DDI	Conditional TdP risk	Low risk DDI
		Lansoprazole, rabeprazole	-	Conditional TdP risk	High risk DDI (major CYP3A4 substrate)
	Antidiarrheals	Racecadotril, loperamide	Low risk DDI	Low risk DDI with racecadotril. loperamide has conditional TdP risk	Low risk DDI
	Prokinetics and laxative	Lactulose, macrogol, magnesium hydroxide, scopolamine-butylbromide	Low risk DDI	Low risk DDI	-
		Cinitapride, naloxegol	-	-	High risk DDI (major CYP3A4 substrate)
	Antihistamines	Dexchlorpheniramine, cetirizine, loratadine, desloratadine, fexofenadine	Low risk DDI	Low risk DDI	-
		Diphenhydramine, promethazine	Low risk DDI	Conditional to possible TdP risk	Low risk DDI

		Ebastine, rupatadine	-	-	High risk DDI (major CYP3A4 substrate)
Hypertension and congestive heart failure treatment	Sartans	All	Low risk DDI	Low risk DDI	
		Losartan	-	-	High risk DDI (major CYP3A4 substrate). Losartan: NTI
	ACE inhibitors	Enalapril, captopril, fosinopril, ramipril, quinapril	Low risk DDI	Low risk DDI	-
	Beta blockers	All	Low risk DDI	Low risk DDI	-
		Bisoprolol	-	-	High risk DDI (major CYP3A4 substrate). Bisoprolol: NTI
	Calcium channel blockers	Clevidipine	Low risk DDI	Low risk DDI	-
		All dihydropyridines	-	-	High risk DDI (major CYP3A4 substrate). Nifedipine and nicardipine moderate CYP3A4 inhibitors.
		Non dihydropyridines (verapamil, diltiazem)	-	-	High risk DDI (major CYP3A4 substrate). Verapamil moderate CYP3A4 inhibitor
	Diuretics	Loop diuretics (furosemide, torsemide, hydrochlorothiazide, indapamide)	Low risk DDI	Conditional TdP risk	Low risk DDI
		Potassium sparing diuretics (amiloride, triamterene, spironolactone)	Low risk DDI	Low risk DDI	-
		Potassium sparing diuretics (eplerenone)	-	-	High risk DDI (major/sensitive CYP3A4 substrate)
Glucose-lowering treatment	Sulfonylureas	Glicazide, glibenclamide, glisentide, glipizide, gliquidone	Low risk DDI	Low risk DDI	-
	Alpha glycosidase inhibitors	Acarbose, miglitol	Low risk DDI	Low risk DDI	-
	GLP-1	Albiglutide, dulaglutide, exenatide, liraglutide, lixisenatide	Low risk DDI	Low risk DDI	-

	DPP-4 inhibitors	Vildagliptin, alogliptin, sitagliptin	Low risk DDI	Low risk DDI	-
		Saxagliptin, linagliptin	-	-	High risk DDI (major CYP3A4 substrate)
	SGLT2 inhibitors	Canagliflozin, dapagliflozin	Low risk DDI	Low risk DDI	-
	Biguanides	Metformin	-	-	Risk of competition for the membrane transporters
	Metglinides	Repaglinide	-	-	High risk DDI (major CYP3A4 substrate)
Lipid-lowering treatment	Statins	Pitavastatin	Low risk DDI	Low risk DDI	-
		Fluvastatin, pravastatin, rosuvastatin	-	Unknown risk of QT prolongation	-
		Simvastatin, atorvastatin	-	-	High risk DDI (major CYP3A4 substrate). Both simvastatin and atorvastatin are sensitive and moderate-sensitive
	Fibrates	All	Low risk DDI	Low risk DDI	-
Antiplatelet	COX-1 inhibitors	ASA, triflusal	Low risk DDI	Low risk DDI	-
	PDE-3 inhibitors	Dipyridamole	Low risk DDI	Low risk DDI	-
		Clilostazol	Weak CYP3A4 inhibitor of CYP3A4	-	High risk DDI (major CYP3A4 substrate)
	P2Y antagonists	Ticlopidine, ticagrelor, prasugrel	Ticagrelor is a weak inhibitor of CYP3A4	-	High risk DDI (major CYP3A4 substrate). Ticagrelor: sensitive/NTI
		Clopidogrel	Low risk DDI	Low risk DDI	-
	GP IIb/IIIa antagonists	Abciximab, eptifibatide, tirofiban	Low risk DDI	Low risk DDI	-
	Selective IP Prostacyclin Receptor Agonist	Selexipag	P-gp, BCRP substrate. Minor CYP3A4 substrate	-	-
	PGI2 analogue	Iloprost, epoprostenol sodium	Low risk DDI	Low risk DDI	-

Anticoagulant	Coumarin	Warfarin, acenocoumarol	Low risk DDI	Low risk DDI	-
	Heparin	Heparin and LMWH	Low risk DDI	Low risk DDI	-
	DOACs FIIa inhibitors	Bivalirudina	Low risk DDI	Low risk DDI	-
		Dabigatran	-	Unknown risk of QT prolongation (under review)	P-gp substrate
	DOACs Fxa inhibitors	Edoxaban	P-gp substrate. Minor CYP3A4 substrate	-	-
		Apixaban, rivaroxaban	-	-	High risk DDI (major CYP3A4 substrate). P-gp and BCRP substrates. Rivaroxaban: moderate-sensitive substrate
Analgesics	Non-opioid	All	Low risk DDI	Low risk DDI	-
		Parecoxib	-	-	Major CYP3A4 substrate.
		Ergotamine and dihydro-ergotamine	-	-	High risk DDI (major CYP3A4 substrate). Ergotamine: NTI
	Opioid	Morphine, hydromorphone, tapentadol, Codeine	Low risk DDI	Low risk DDI	-
		Tramadol, buprenorphine, oxycodon	-	Possible for tramadol and buprenorphine	Major CYP3A4 substrate
		methadone, fentanyl	-	Known TdP risk for methadone	High risk DDI (major CYP3A4 substrate). Fentanyl: NTI. Consider dose adjustment
Anticonvulsivants and Adjuvants	-	Pregabalin, gabapentin, levetiracetam, lamotrigine, topiramate, baclofen, clonidine, octreotide, alendronate, zoledronate, denosumab	Low risk DDI	Low risk DDI	-
	-	Lacosamide, valproic acid, prednisone, methylprednisolone, hydrocortisone, fextromethorphan	Low risk DDI	Low risk DDI	-

	-	Dexamethasone, zonisamide, oxcarbazepine, ketamine	Dexamethasone and Oxacarazepine are weak CYP3A4 inducers	Conditional TdP risk	Except oxcarbazepine major CYP3A4 substrate.
	-	Carbamazepine, phenobarbital, phenytoin	Strong CYP3A4 inducers	Conditional to known TdP risk	Carbamazepine and phenytoin are substrates of CYP3A4, respectively major and minor
Antidepressants	-	Duloxetine, desvenlafaxine, vortioxetine	Low risk DDI	Low risk DDI	-
	-	Paroxetine, sertraline, fluoxetine	-	Conditional TdP risk.	-
	-	Trazodone, mirtazapine, venlafaxine, citalopram, escitalopram	-	-	High risk DDI (major CYP3A4 substrate). Citalopram and escitalopram: NTI
Antipsychotic	-	Olanzapine, amisulpride	Low risk DDI	Conditional to possible TdP risk. However, the risk is dose dependent	-
	-	Paliperidone, risperidone, asenapine, perphenazine, clozapine, quetiapine	-	Conditional to possible TdP risk	High risk DDI. Quetiapine: major CYP34 substrate and NTI
	-	Sulpiride, chlorpromazine, levopìmepromazine, ziprasidone, aripiprazole, haloperidol, pimozide	Ziprasidone moderate CYP3A4 inhibitor.	Conditional to known TdP risk	High risk DDI. Pimozide: major CYP34 substrate and NTI
Anxiolytics and hypnotics	-	Lorazepam, lormetazepam, clotiazepam, bromazepam, clobazam	Low risk DDI. Clobazapam weak CYP3A4 inducer	Low risk DDI	-
	-	Diazepam, clorazepate, clonazepam, midazolam, flurazepam, alprazolam, zolpidem, zopiclone	Alprazolam weak CYP3A4 inhibitor	-	-
